# Comparative evaluation of ACetic - MEthanol high salt dissociation approach for single-cell transcriptomics of frozen human tissues

**DOI:** 10.3389/fcell.2024.1469955

**Published:** 2025-01-07

**Authors:** Marina Utkina, Anastasia Shcherbakova, Ruslan Deviatiiarov, Alina Ryabova, Marina Loguinova, Valentin Trofimov, Anna Kuznetsova, Mikhail Petropavlovskiy, Rustam Salimkhanov, Denis Maksimov, Eugene Albert, Alexandra Golubeva, Walaa Asaad, Lilia Urusova, Ekaterina Bondarenko, Anastasia Lapshina, Alexandra Shutova, Dmitry Beltsevich, Oleg Gusev, Larisa Dzeranova, Galina Melnichenko, Ildar Minniakhmetov, Ivan Dedov, Natalya Mokrysheva, Sergey Popov

**Affiliations:** ^1^ Endocrinology Research Centre, Institute of Personalized Medicine, Moscow, Russia; ^2^ Graduate School of Medicine, Juntendo University, Bunkyo-ku, Japan; ^3^ Life Improvement by Future Technologies (LIFT) Center, Moscow, Russia; ^4^ Faculty of Medicine, Lomonosov Moscow State University, Moscow, Russia; ^5^ Regulatory Genomics Research Center, Institute of Fundamental Medicine and Biology, Kazan Federal University, Kazan, Russia

**Keywords:** ScRNA-seq, ACME HS, cryopreservation, dissociation, fresh-frozen tissue, fixed single cells, human endocrine glands

## Abstract

Current dissociation methods for solid tissues in scRNA-seq studies do not guarantee intact single-cell isolation, especially for sensitive and complex human endocrine tissues. Most studies rely on enzymatic dissociation of fresh samples or nuclei isolation from frozen samples. Dissociating whole intact cells from fresh-frozen samples, commonly collected by biobanks, remains a challenge. Here, we utilized the acetic-methanol dissociation approach (ACME) to capture transcriptional profiles of individual cells from fresh-frozen tissue samples. This method combines acetic acid-based dissociation and methanol-based fixation. In our study, we optimized this approach for human endocrine tissue samples for the first time. We incorporated a high-salt washing buffer instead of the standard PBS to stabilize RNA and prevent RNases reactivation during rehydration. We have designated this optimized protocol as ACME HS (ACetic acid-MEthanol High Salt). This technique aims to preserve cell morphology and RNA integrity, minimizing transcriptome changes and providing a more accurate representation of mature mRNA. We compared the ability of enzymatic, ACME HS, and nuclei isolation methods to preserve major cell types, gene expression, and standard quality parameters across 41 tissue samples. Our results demonstrated that ACME HS effectively dissociates and fixes cells, preserving cell morphology and high RNA integrity. This makes ACME HS a valuable alternative for scRNA-seq protocols involving challenging tissues where obtaining a live cell suspension is difficult or disruptive.

## 1 Introduction

Recent advances in single-cell RNA sequencing (scRNA-seq) have dramatically expanded our understanding of the cellular complexity and heterogeneity of human tissues, including endocrine glands (Zhang et al., 2020; Kameneva et al., 2022; Dong et al., 2020). However, further progress in this field struggled with the incomplete molecular characterization of the particular cell types being responsible for the functional complexity of human endocrine tissues. One of the most problematic issues in the scRNA-seq profiling of human tissues that significantly impacts the biological relevance of the ultimate data is the sample preparation step. In that, the most commonly used immediate processing of freshly isolated tissues is extremely poorly integrated in the clinical logistics of tedious surgical procedures and subsequent surgical pathology assessments. This issue is particularly important in the context of the inherent problems in obtaining the high-quality single cell suspensions from solid tissues, requiring complex disaggregation/dissociation steps while preserving the high cellular yield and viability, unbiased cellular contents, transcriptional profiles, cellular states, etc. The suboptimal procedures used for tissue collection, storage, and especially disaggregation/cell dissociation, which involve excessive mechanical stress, suboptimal temperature conditions, prolonged enzymatic digestion, and the loss of the original tissue context, have been shown to significantly distort the resulting scRNA-seq data and may lead to cell type misclassification (Slyper et al., 2020; van den Brink et al., 2017).

Since the late 1970s, when the first methods for the disaggregation of solid tissues were described (Waymouth, 1974; Cerra et al., 1990), a variety of protocols utilizing mechanical, enzymatic, and chemical methods of dissociation (and combinations thereof) have emerged (Cunningham, 2010; Denisenko et al., 2020). Probably the most popular approach for obtaining single cell suspensions for scRNA-seq is enzymatic digestion implying the incubation of gross tissue samples with various proteases at 37°C as a key step. However, a number of studies have demonstrated that the employment of these techniques is associated with the profound activation of the stress signaling pathways and increased cell death, resulting in a significant bias in the scRNA-seq profiles (van den Brink et al., 2017; Lake et al., 2016). Such undesirable effects may be largely diminished via employment of the so-called “cold” dissociation techniques with a cold active protease (6°C) (O’Flanagan et al., 2019), however, at the cost of less efficient target cell dissociation (Deleersnijder et al., 2021). An alternative strategy to prevent shifts in transcriptional profiles is the use of general transcription inhibitors, such as Actinomycin D (ActD) (Lai et al., 2019). ActD has been shown to effectively mitigate artifact formation and block transcriptional changes during the dissociation of neuronal tissue, thus preserving the integrity of the transcriptional profile (Wu et al., 2017). However, the routine use of such inhibitors is limited by their toxicity, which depends on both concentration and cell type.

Furthermore, the enzymatic dissociation of normal and diseased endocrine tissues may be particularly challenging due to a number of confounding structural issues, such as a high lipid content in the normal adrenal cortex and adrenocortical neoplasia, or extensive stromal/capsular fibrosis and calcifications in the well-differentiated thyroid tumors. Single-nucleus RNA sequencing (snRNA-seq) protocols being compatible with the use of the fresh-frozen tissue samples may be successfully employed to overcome these limitations (Lake et al., 2016; Schmøkel et al., 2023); however, at the cost of the loss of a significant amount of the mature cytoplasmic mRNA, resulting in a lower coverage and poor representation of the rare cell types (Thrupp et al., 2020).

Each of the abovementioned approaches contributes to the emergence of a variety of artifacts related to the distortion of the transcriptional profiles of individual cells (Deleersnijder et al., 2021), an issue that must be explicitly addressed in the process of the ultimate data analysis (Massoni-Badosa et al., 2020). For example, the immediate-early response genes (e.g., the members of the *FOS* and *JUN* gene families) are primary candidates for changing their expression during single-cell dissociation at 37°C. Artifactual changes in gene expression patterns were investigated by comparing the transcriptional profiles of cryopreserved and living cells or methanol-fixed and living cells obtained from tissue dissociation using cold-active protease and enzymatic digestion at 37°C. This study showed that cold-active proteases dramatically reduce the number of scRNA-seq artifacts in the mouse kidney (Adam et al., 2017). However, addressing of these issues remains rather fragmentary and limited to certain tissue types and protocols, so numerous artifacts still need to be confidently addressed.

Many of the limitations of the currently employed techniques may be potentially overcome via simultaneous tissue dissociation and cell fixation, the procedure being capable of maintaining a high RNA Integrity Number (RIN) while minimizing the sample preparation-related distortions in the transcriptional profiles. Recently, García-Castro et al. (2021) introduced such a protocol whose prototype may be tracked back to the end of the 19th century (Canmillo Schneider, 1890; David, 1973), when Schneider reported the so-called “maceration” technique. In its contemporary variant, named ACME (ACetic (acid)-MEthanol), this technique reportedly produces quality suspensions of fixed single cells from planarians, *D. melanogaster*, *D. rerio*, and *M. musculus* tissues, where suspensions maintaining the high-integrity RNA may be further successfully cryopreserved using DMSO (García-Castro et al., 2021).

Here we extensively optimized and successfully implemented the unique ACME High Salt (ACME HS, see *Methods*) protocol for the single-cell transcriptomics analysis of human endocrine tissues represented by tumors arising from the adrenal medulla, adrenal cortex, pituitary gland, and thyroid follicular cells. Additionally, the incorporation of the high-salt washing buffer (3xSSC*) in ACME HS protocol instead of the standard PBS allowed us to stabilize RNA and prevent RNases reactivation during rehydration. The increased ionic strength of 3xSSC* buffer affects protein solubility and interactions, creating an environment that effectively impedes RNases function. Higher salt concentrations can alter protein structures, thereby reducing enzymatic activity and making it difficult for RNases to function effectively (Baba et al., 2017). We also compared our modified ACME HS and enzymatic dissociation methods for scRNA and nuclei isolation for snRNA profiling in terms of the number of the cells/nuclei recovered, RNA integrity, aligning of the resulting scRNA/snRNA data with the reference organ-specific profiles, and representation of specific cell types.

We clearly demonstrated that scRNA profiling of single cell suspensions obtained using ACME HS and enxymatic dissociation methods significantly outperformed snRNA profiling in terms of marker genes expression analysis while demonstrating in-between comparable performances in virtually all implemented analyses. Additionally, the modified ACME HS protocol allows successful cryopreservation of dissociated/fixed cells without sacrificing the mRNA yield and integrity. To our knowledge, this is the first report on successful implementation of the ACME HS technique in primary human tissues, and we believe that this protocol should significantly promote the scRNA studies in humans that are to be explicitly compliant with the real-life infrastructure and logistics of the surgical care centers.

## 2 Materials and methods

### 2.1 Tissue sampling

Twenty-eight human adrenal gland neoplasms (including 12 adrenocortical tumors and 16 adrenal medullary tumors), nine thyroid carcinomas, and 12 pituitary neuroendocrine tumors (PitNETs) were acquired from the Endocrinology Research Centre, Moscow, Russia (Supplementary Table S1). In all patients, tumor specimens were definitively diagnosed by imaging, surgery, and histopathological examination. Each study participant gave written informed consent. In addition, single cells were isolated from fresh and fresh-frozen adrenal medullary tumor, adrenocortical tumor, thyroid carcinoma, and PitNET samples. After sampling, the tissues were placed in a cold Tissue Storage Solution (Miltenyi Biotec) pending dissociation. The fresh-frozen samples were stored at −80°C until processed.

### 2.2 ACME HS dissociation

After tissue sampling, fresh-frozen adrenal gland neoplasms (200–250 mg), thyroid carcinoma (200–250 mg), or PitNET samples (5–10 mg) were thoroughly minced on ice. The minced tissue was then immediately added to an ACME solution consisting of 15% methanol (Sigma-Aldrich, United States), 0.1M glacial acetic acid (Sigma-Aldrich, United States), 0.1M glycerol (Thermo Fisher Scientific, United States), 0.1M N-acetyl cysteine (NAC) (Sigma-Aldrich, United States), and RNase-free water (QIAGEN, United States), achieving a total volume of 10 mL in a 15 mL Falcon tube. NAC (Sigma-Aldrich, United States) is included in the buffer to remove mucus and fatty lipids from cells while providing protection against oxidative damage. Glycerol (Thermo Fisher Scientific, United States) is added to stabilize cellular membranes, reduce mechanical stress on cells, and enhance the preservation of cellular structures during dissociation.

The samples were dissociated at room temperature for 1 hour on a shaker set to 35 rpm, using vertical platform rotation. During this incubation, the mixture was gently pipetted 2 to 4 times with 5 mL pipette tips to ensure thorough mixing. Following incubation, the samples were centrifuged at 1,000×g for 5 min at 4°C to remove the ACME solution, after which they were kept on ice to prevent RNA degradation. The supernatant was carefully discarded, and 2–4 mL of cold, 3xSSC* (saline-sodium citrate) buffer (composed of 3xSSC (Invitrogen, Thermo Fisher Scientific, United States), 40 mM DTT (Wuhan Servicebio Technology, China), 1% BSA (Thermo Fisher Scientific, United States), and RNase-free water) was added to the cell pellet, along with 0.5 U/µL of the RNase inhibitor RiboLock (Thermo Fisher Scientific, United States), and the cells were resuspended. The homogenate was sequentially filtered through pre-wetted (with 500 µL of 3xSSC*) 70 μm and 40 μm filters (Miltenyi Biotec, Germany) into a 15 mL tube. Following filtration, the samples were centrifuged at 1,000×g for 7 min at 4°C to separate the cells from debris. The supernatant was then carefully removed to avoid disturbing the pellet. Finally, the pellet was resuspended in 1–2 mL of cold 3xSSC* buffer to maintain cellular integrity and prepare the cells for subsequent analysis.

ACME solution and 3xSSC* buffer were freshly prepared. The 3xSSC* buffer was kept on ice immediately after preparation and prior to use. The concentrations of all stock solutions used before buffer and solution preparation were as follows: 1M glacial acetic acid, 1M glycerol, 1M NAC, 1M DTT, 100% methanol, 30% BSA, and 20xSSC stock solution. Additionally, 1M DTT and 30% BSA were stored at −20°C, 1M NAC at +4°C, and the other stock solutions were stored at room temperature.

### 2.3 Enzymatic dissociation

Approximately 200–250 mg of fresh adrenal gland neoplasm, thyroid carcinoma, or 5–10 mg of PitNET samples were washed in HBSS (Gibco, Thermo Fisher Scientific, United States), thoroughly minced on ice, and placed in dissociating solution at 37°C with gentle pipetting every 5 min. Adrenal gland neoplasm samples were dissociated using 25–30 µL of enzyme D from the Multi Tissue Dissociation Kit (Miltenyi Biotec, Germany) in 870 mM HBSS (Gibco, Thermo Fisher Scientific, United States), 10% FBS (HyClone, Thermo Fisher Scientific, United States), and 20 mM HEPES (Gibco, Thermo Fisher Scientific, United States) for 20–30 min. For the thyroid carcinoma samples, we used 30–35 µL of the same enzyme and conditions for 20–30 min. PitNET samples were dissociated with 8–10 µL of enzyme D under identical conditions for 7–15 min. The resulting homogenate was filtered through a pre-wetted 70 μm cell culture filter (Miltenyi Biotec, Germany) into 3–5 mL of Wash Buffer (1xDPBS without Ca & Mg (Capricorn Scientific, Germany) containing 10% FBS (HyClone, Thermo Fisher Scientific, United States), 20 mM HEPES (Gibco, Thermo Fisher Scientific, United States), and 6 mM glucose (Gibco, Thermo Fisher Scientific, United States) and then centrifuged for 5 min at 300xg at 4°C. For samples with high blood and debris content, the red blood cells were lysed using Red Blood Cell Lysis Solution (Miltenyi Biotec, Germany), and dead cells were removed with Dead Cell Removal Kit (Miltenyi Biotec, Germany). The cells were counted and assessed for viability using trypan blue staining on Countess 3 (Thermo Scientific, United States). After all, the pellet was resuspended in a Wash Buffer volume of 100–400 μL, depending on the pellet size.

### 2.4 Nuclei isolation

Nuclei were isolated from fresh-frozen adrenocortical tumor, adrenal medullary tumor, and PitNET specimens. Fresh-frozen tissue samples were thoroughly minced on ice and placed into a gentleMACS C tube (Miltenyi Biotec, Germany) with 2 mL ice-cold Hypotonic lysis buffer (10 mM HEPES pH7.2 (Gibco, Thermo Fisher Scientific, United States), 5 mM MgCl_2_ (Sigma-Aldrich, Merck, United States) 10 mM NaCl (Sigma-Aldrich, Merck, United States), and 1% NP40 Surfact-Amps (Thermo Fisher Scientific, United States)). GentleMACS C tubes were then placed on the gentleMACS Dissociator (Miltenyi Biotec, Germany) and the samples were homogenized by running the program h_mito_01, and then incubated on ice for 10 min. After repeating the homogenization step, 2 mL of Isotonic buffer (10 mM HEPES pH7.2 (Gibco, Thermo Fisher Scientific, United States), 5 mM MgCl_2_ (Sigma-Aldrich, Merck, United States),10 mM NaCl (Sigma-Aldrich, Merck, United States), and 500 mM sucrose (MilliporeSigma, Merck, United States)) was added to the lysates, mixed by pipetting, filtered through a pre-wetted 70 μm cell culture filter (Miltenyi Biotec, Germany) with Isotonic wash buffer (10 mM HEPES pH7.2 (Gibco, Thermo Fisher Scientific, United States), 5 mM MgCl_2_ (Sigma-Aldrich, Merck, United States), 10 mM NaCl (Sigma-Aldrich, Merck, United States), and 250 mM sucrose (MilliporeSigma, Merck, United States)) and centrifuged for 5 min at 1000xg at 4°C. Then, we carefully removed the supernatant, resuspended the pellet in 1 mL of 1xDPBS without Ca & Mg (Capricorn Scientific, Germany) with 1% BSA (Thermo Fisher Scientific, United States), and filtered it through a prewetted 30 μm cell culture filter (Miltenyi Biotec, Germany). The pellet was then resuspended in the same 1xDPBS without Ca & Mg, containing 1% BSA, in a volume of 100–400 μL, depending on the pellet size.

### 2.5 RNA extraction and quality assessment

To evaluate the RIN of the samples depending on the duration of their storage, we isolated RNA from cell suspensions prepared by the ACME HS and enzymatic dissociation methods. We isolated RNA from freshly obtained ACME HS and enzyme-dissociated cells immediately after dissociation (day 0), as well as from cryopreserved ACME HS cells and frozen enzyme-dissociated cells at intervals of 1, 3, 7, 14, and 28 days. RNA extractions were performed using an AllPrep DNA/RNA Mini Kit (QIAGEN, United States) following the manufacturer’s protocol. RNA quality was assessed on an Agilent 5,200 Fragment Analyzer (Agilent Technologies, United States), using the Agilent HS RNA (15NT) kit (Agilent Technologies, United States).

### 2.6 Immunocytochemistry

Immunocytochemistry was performed for the markers CYP11B1 and TSHR to identify adrenocortical and thyroid cells. Initially, membranes of enzyme-dissociated cells were permeabilized with 100 ul of permeabilization enzyme (10X Genomics, United States) for 20 min at 37°C. After that, the ACME HS and enzyme-dissociated cells were blocked in 3% BSA (Thermo Fisher Scientific, United States) for 20 min. The cells were incubated in the antibody diluent buffer (Abcam, United States) of the primary polyclonal Anti-CYP11B1 antibody (ab197908, Abcam, United States) and the TSH Receptor monoclonal antibody (4C1) (MA5-16519, Invitrogen, Thermo Fisher Scientific, United States) at a dilution of 1:200 for 1 h at 4°C. The cells were washed in antibody diluent buffer before being incubated for 1 h at 4°C with the secondary antibody AlexaFluor 594 (ab150080, Abcam, United States) or AlexaFluor 594 (ab150116, Abcam, United States) at a dilution of 1:500, respectively. After repeating the washing step, the cells were stained with Hoechst 33342 (BD Pharmingen, United States). Visualization of the antigen-antibody complexes was performed using the Olympus FV3000 Scanning Confocal Microscope (Olympus corporation, Japan), 60х, scan size 2048x2048, VBF (Variable Barrier Filter) mode, dye: Hoechst 33342 and Alexa Fluor 594.

### 2.7 Methanol fixation and ACME HS cryopreservation

For methanol fixation, 200 µL of previously prepared enzyme-dissociated cells in Wash buffer (see *Methods, Enzymatic dissociation*) supplemented with 0.5 u/µl of the RNase Inhibitor RiboLock (Thermo Fisher Scientific, United States) was used. Ice-cold 100% methanol in a volume 800 µL (Sigma-Aldrich, United States) was added dropwise to the cells while gently vortexing to prevent cell clumping. The fixed cells were then stored at −80°C.

For ACME HS cryopreservation, 900 µL of cell suspension in 3xSSC* buffer (see *Methods, ACME HS dissociation*) was mixed with 10% DMSO (Thermo Fisher Scientific, United States) and cryopreserved. The preserved cells were stored at −80°C.

### 2.8 Flow cytometry

ACME HS and enzyme-dissociated cells isolated from adrenal gland neoplasm, thyroid carcinoma, and PitNET samples were transferred into 1xDPBS without Ca & Mg (Capricorn Scientific, Germany) with 0.1% FBS (HyClone, Thermo Fisher Scientific, United States) at a final concentration of 10^6^ cells/ml. Next, 2 µM of 5.6-carboxyfluorescein diacetate succinimidyl ester or CFSE (BD Biosciences, United States) was added to the cells and incubated for 5 min at 370C. Then, the cells were washed twice with 10 volumes of cold 1xDPBS without Ca & Mg (Capricorn Scientific, Germany), and stained with PI (10 μg/mL) in 0.5 mL of PI/RNase staining buffer (BD Biosciences, United States) for 30 min at room temperature in the dark.

Flow cytometric analysis was performed on a NovoCyte 2060R system (Agilent Technologies, United States) equipped with two lasers, including a laser tuned at 488 nm to excite CFSE and PI, and the standard set of detectors for green fluorescence of CFSE and red fluorescence of PI. Program compensation was used to correct spectral spillover. Fluidics and optics were calibrated with NovoCyte QC particles. The threshold was set at FSC-H. Samples were run at the lowest flow rate. At least 10,000 events were analysed. Deconvolution of the DNA histograms was performed with the instrument Software NovoExpress (Agilent Technologies, United States).

### 2.9 Preparation of the cell suspensions for loading on the 10x chromium controller

ACME HS-cryopreserved and methanol-fixed cells were thawed and centrifuged at 2000×g for 5 min at 4°C to remove the 3xSSC*/DMSO and methanol. The resulting pellet was then resuspended in cold 3xSSC* buffer to a concentration of approximately 2000 cells or nuclei/µl.

### 2.10 10x Genomics sn/scRNA-seq library preparation

Single cells or nuclei were captured and barcoded, and cDNA libraries were generated using the Chromium Next GEM Single Cell 3ʹGEM, Library & Gel Bead Kit v3.1 (10X Genomics, United States). For each sample, approximately 10,000 cells or nuclei (∼2000 cells or nuclei per 1µL, as calculated using the cell suspension volume calculator from 10X Genomics) in cold 3xSSC* buffer were mixed with RT-PCR master mix and immediately loaded, along with Single-Cell 3′Gel Beads and Partitioning Oil, into a Chromium Chip G. cDNA and gene expression libraries were then generated following the manufacturer’s instructions (10x Genomics, United States). cDNA and gene expression libraries were quantified using the Qubit dsDNA HS Assay Kit (Thermo Fisher Scientific, United States), and fragment sizes were assessed with the Agilent 5,200 Fragment Analyzer (Agilent Technologies, United States) using the DNA HS (1–6,000 bp) Kit (Agilent Technologies, United States). Final libraries were then multiplexed and sequenced on an Illumina NovaSeq 6,000 platform (Illumina, United States) with the S4 Reagent Kit v1.5 (200 cycles) (Illumina, United States).

### 2.11 Adrenal, thyroid and pituitary glands single-cell transcriptomic analysis

The raw sequenced reads were processed with 10X Cell Ranger (v6.1.1). Default Cell Ranger quality check measurements were used for further comparison methods through the Wilcoxon test. The expression matrixes for the filtered cells were submitted to Seurat (Hao et al., 2023) (v4.9.9 and v5.0.0) for basic analysis, including scaling and normalization. Cell filtering based on gene/molecule dependency was done by pagoda2 (pagoda2, 2023) (v1.0.11). For the pagoda2 package, we utilized a minimum cell size of 1,000 and a maximum cell size of 50,000. The p.level was set as the minimum between 0.001 and 1/(number of columns in countMatrix). Doublets and ambient RNA content were calculated with scrublet (Wolock et al., 2019) (v0.2.3), SoupX (Young and Behjati, 2020) (v1.6.2) and decontX (Yang et al., 2020) (v3.18), respectively, with default settings. Specifically, for the Scrublet package, the following hyperparameters were applied: the number of doublets simulated relative to the number of observed transcriptomes was set to 2.0. The estimated doublet rate for the experiment was 0.05, with a standard deviation of 0.02. The sampling rate for UMIs when creating synthetic doublets was set to 1.0. The number of principal components used to embed the transcriptomes before k-nearest-neighbor graph construction was fixed at 30. For the SoupX package, the term frequency–inverse document frequency (tf-idf) minimum was established at 1. We only included genes that were at or above the 0.9 expression quantile. The maximum number of markers was limited to 100, and the contamination range was set to c (0.01, 0.08). The maximal false discovery rate was fixed at 0.2, while the mode of the gamma distribution prior on the contamination fraction was set to 0.05, with a standard deviation of 0.1. For the decontX package, the maximum iterations of the expectation-maximization (EM) algorithm were set to 500. We defined concentration parameters for the Dirichlet prior for the contamination in each cell (native and contamination counts) as c (10, 10), updating these values with fit_dirichlet during each iteration. The convergence threshold for the EM algorithm was established at 0.001, and the log likelihood was set to 10. We selected 5,000 variable genes for dimensionality reduction prior to clustering, with the clustering resolution parameter used in ‘dbscan’ to estimate broad cell clusters set to 1. A seed value of 12,345 was provided to seed_with for reproducibility. The means for doublets and ambient RNA values per sample were compared between sample preparation methods (ACME HS, enzymatic, nuclei). Major cell types were identified by the label propagation function using Conos (conos, 2023) (v1.5.0) and reference datasets (Jansky et al., 2021; Kildisiute et al., 2021; Han et al., 2020; Torgersen et al., 1995; Zhang et al., 2022). Sample integration was conducted by applying RunHarmony on preprocessed Seurat objects (Korsunsky et al., 2019). Velocity analysis was performed with Velocyto (v0.17) on 1,000 cells subset per sample and visualized using Velocyto.R (La Manno et al., 2018) (v0.6) on integrated embeddings. In addition, we applied scVelo (v0.3.2) (Bergen et al., 2020) on whole samples in order to obtain confidence levels of velocity estimations and pseudotime. Differential expression analysis and cell cycle phase predictions were processed with Seurat (v5.0.0). Significance of phases enrichment over cell types calculated with chi-squared test. A functional enrichment test was performed for differentially expressed genes with clusterProfiler (Wu et al., 2021) and wiki pathways (Agrawal et al., 2023) as reference databases.

### 2.12 Analysis of stress and cell death gene signatures

We used the PercentageFeatureSet function with default parameters from the Seurat package to evaluate the impact of various methods—ACME HS, enzymatic dissociation, and nuclei isolation—on cellular stress, focusing on their potential to induce stress or favor necrosis or apoptosis in cells. This function calculates the percentage of total counts assigned to a specified set of genes. For the apoptosis signature, we curated a gene signature encompassing *CASP3, BAX, BAD, BID, APAF1, TP53, FAS, TNFRSF10B, CYCS, BCL2,* and *AIFM1*(Kiraz et al., 2016). Meanwhile, for the necrosis signature, we selected *HMGB1* (Nikoletopoulou et al., 2013)*, ATP5F1A, CALR, ARHGAP45, S100A8, S100A9, NAMPT, ANXA1, KRT18, TNF,* and *AGER* genes (Kanehisa et al., 2021; Kanehisa, 2019). We assessed various modalities of cell stress, including oxidative stress, cellular senescence, DNA damage, heat shock, and the unfolded protein response. The oxidative stress signature was constructed using *NFE2L2, KEAP1, SOD1, CAT, HMOX1, GCLC, GCLM, NQO1,* and *PRDX1* genes (Kanehisa et al., 2021; Kanehisa, 2019). The markers of cellular senescence included *CDKN1A, CDKN2A, IGFBP3, GADD45A, CCND1, CDKN2B, IL1A, IL1B, IL6, IL10, HMGA1, HMGB2,* and *UBB*(Kanehisa et al., 2021; Kanehisa, 2019). DNA damage signatures comprised *TP53, BRCA1, CHEK2, ATM, RAD51, RPA1, MDM2, ATR,* and *XRCC5*(Kanehisa et al., 2021; Kanehisa, 2019). For the heat shock signature, we considered the HSP family genes *HSPB, HSPG2, HSPB11, HSPA6, HSPD1, HSPE1, HSPBAP1, HSPA4L, HSPB3, HSPA4, HSPA9, HSPA1L, HSPA1A, HSPA1B, HSP90AB1, HSPB1, HSPA5, HSPA14, HSPA14.1, HSPA12A, HSPB2, HSPA8, HSP90B1, HSPB8, HSPH1, HSPA2, HSP90AA1, HSPB9, HSPB6, HSPBP1, HSPA12B,* and *HSPA13*(Yer et al., 2018; Kampinga et al., 2009). Lastly, the unfolded protein response signature included *ATF4, ATF6, XBP1, HSPA5, DDIT3, HERPUD1, DNAJC3, ERN1, ERN2,* and *PDIA6* genes (Kanehisa et al., 2021; Kanehisa, 2019). Using a two-tailed Wilcoxon rank-sum test, we calculated statistically significant differences in the signature scores between the various dissociation methods.

### 2.13 Statistical data analysis

All the data were presented as the means and standard deviations. Statistical significance (assessed by two-tailed t-test and Wilcoxon rank-sum test) is shown in the Figureures: **** (0.0001 < *p* < 0.001), *** (*p* < 0.001), ** (0.001 < *p* < 0.01), * (0.01 < *p* < 0.05), ns - not significant - *p* > 0.05.

## 3 Results

### 3.1 ACME HS-based dissociation of endocrine tumor samples produces fixed cells with high RNA integrity and preserved morphology

Using the adrenocortical tumor sample, we assessed morphology and found that the RNA integrity of ACME HS-dissociated adrenocortical cells was well-preserved. For ACME HS dissociation, a fresh adrenocortical tumor was previously cryopreserved in a biobank and the cell suspension obtained the following day was divided into seven aliquots (six aliquots for the RNA integrity number (RIN) calculation and one for microscopy). Concurrently, enzyme-dissociated cells were obtained from the same fresh adrenocortical tumor and were also divided into seven aliquots.

**FIGURE 1:**
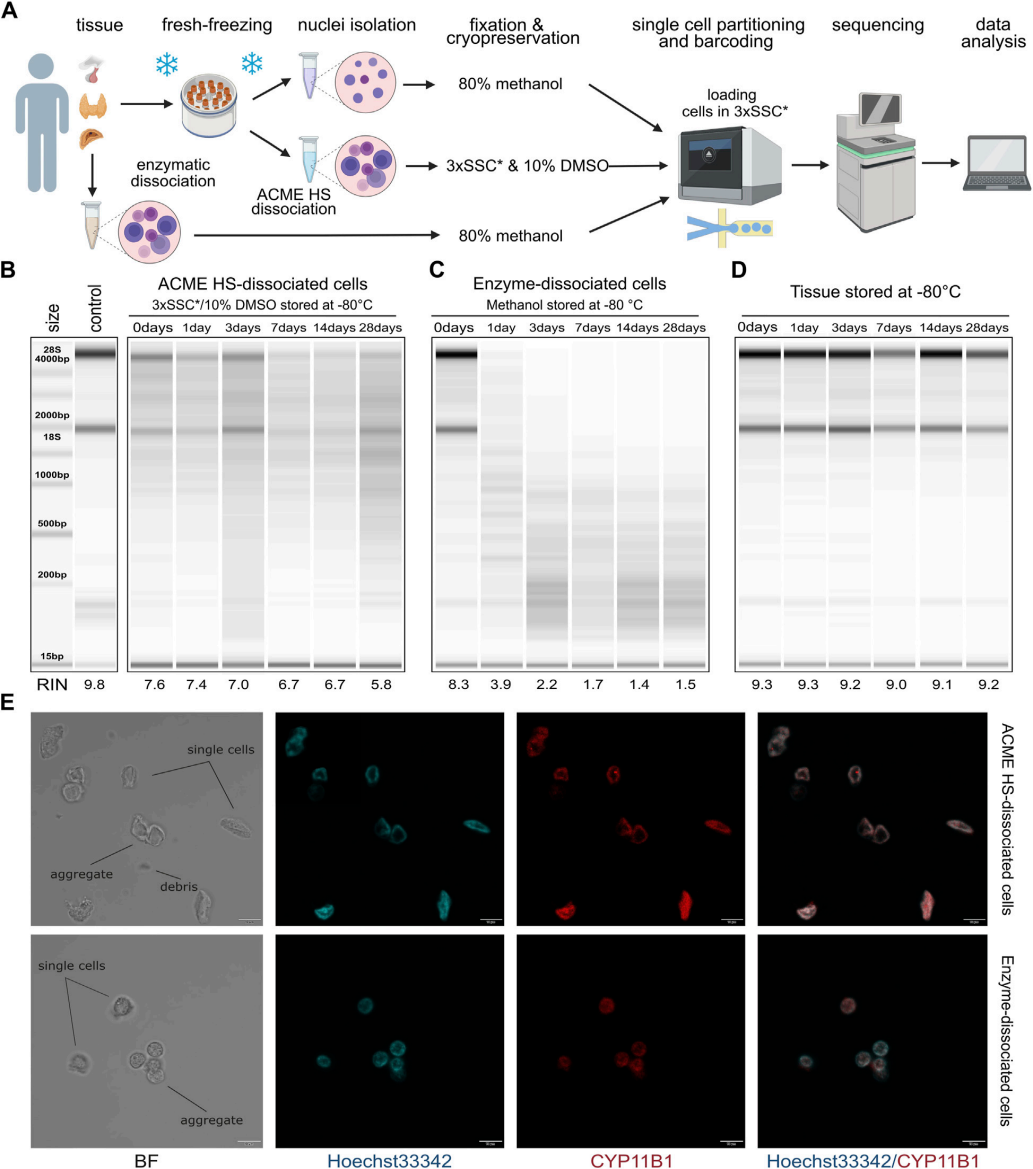
Comparison of RNA integrity, morphology, and storage of ACME HS and enzyme-dissociated adrenocortical cells. **(A)** Schematic representation of a workflow for single-cell or single-nuclei processing and analysis of fresh and fresh-frozen tissues (created with *BioRender.com*). **(B)** Gel image of isolated total RNA from cryopreserved ACME HS-dissociated adrenocortical cells after 1,3,7,14, and 28°days of freezing at −80°C, and of freshly isolated adrenocortical cells kept at +4°C (0°days). **(C)** Gel image of isolated total RNA from adrenocortical cells obtained by enzymatic dissociation and fixed in 80% methanol after 1, 3, 7, 14, and 28°days of freezing at −80°C, and of freshly isolated cells kept at +4°C (0°days). **(D)** RNA integrity of fresh-frozen adrenocortical tumor, from which ACME HS-dissociated **(B)** and enzyme-dissociated **(C)** cells were obtained. **(E).** Bright field (BF) and confocal fluorescence microscopy images of freshly isolated ACME HS and enzyme-dissociated adrenocortical cells stained with Hoechst 33,342 (blue) and anti-CYP11B1 antibody (red), showing single cells, aggregates, and debris.

Single cells were isolated from tissue samples using both ACME HS and enzymatic dissociation methods, freshly and followed by cryopreservation in 3xSSC*10% DMSO or methanol cell fixation, respectively (Figure 1A; Supplementary Table S1). The key adaptations of the ACME HS method included supplementing the solution with 0.1M NAC and adding extra washing steps for isolated cells using cold high-salt 3xSSC* buffer. ACME dissociation was performed for approximately 1 hour on a rotator at room temperature, with periodic pipetting. Afterward, the ACME solution was removed, and the pellet was washed using a two-step procedure with cold 3xSSC* (see *Methods* for details).

Total RNA extracted from freshly prepared cell suspensions (0 days) indicated that the RINs for cells obtained through the ACME HS dissociation method and enzymatic digestion were similar, at 7.6 and 8.3, respectively (Figures 1B, C). Similar RINs scores of RNA were found for cells obtained from adrenal medullary tumor, thyroid carcinoma and pituitary neuroendocrine tumor (PitNET) samples (Supplementary Figure S1). The obtained RIN scores were compared with the RIN score (9.8) of undissociated adrenocortical tumor (control) (Figure 1B).

Next, we visualized freshly prepared dissociated adrenocortical cells (0 days) using bright field and confocal microscopy. The cells maintained their morphology, displaying minimal aggregation and debris (Figure 1E). Additionally, microscopy was conducted for thyroid cells and one replicate of adrenocortical cells. (Supplementary Figure S2B). To identify adrenocortical cells, we stained the fixed cells with Hoechst 33342 and an anti-CYP11B1 (11β-hydroxylase) antibody conjugated with Alexa Fluor 594 (Figure 1E; Supplementary Figure S2B). CYP11B1 is localized in the inner mitochondrial membrane and is normally expressed in the zona fasciculata of the human adrenal cortex (Duparc et al., 2022). We observed intense immunofluorescence of CYP11B1 (red) in ACME HS-dissociated adrenocortical cells, in contrast to cells isolated using enzymatic digestion. This difference could be attributed to the extended permeabilization of cell membranes with methanol during the ACME HS protocol. The voids observed in the nuclei of adrenocortical stained cells are likely associated with their functional ability to efflux the DNA binding dye Hoechst 33342, resulting in the so-called side population (SP) (Crowley et al., 2016, p. 33). Thyroid follicular cells were also visualized by staining fixed cells with Hoechst 33342 and an anti-TSHR (TSH receptor) antibody conjugated with Alexa Fluor 594 (Supplementary Figure S2B).

### 3.2 ACME HS-dissociated cells can be cryopreserved and stored

A key disadvantage of commonly used enzymatic digestion methods is that freshly isolated tissues are extremely poorly integrated in the clinical logistics of surgical procedures. In contrast, the method we used enabled the freezing of fresh tissue samples before ACME HS dissociation. By combining tissue dissociation and cell fixation, the ACME HS method preserves cells within their context. Furthermore, the resulting suspensions can be cryopreserved for subsequent analysis (García-Castro et al., 2021). To confirm that cryopreservation of ACME HS-dissociated cells in 3xSSC* with 10% DMSO maintains RNA integrity, we evaluated the RNA quality in cells after different durations of cryopreservation.

We sequentially extracted total RNA from six aliquots of ACME HS (Figure 1B) and six aliquots of enzyme-dissociated (Figure 1C) cells obtained from the same adrenocortical tumor at various time intervals: immediately after preparing the single-cell suspension (0 days) and after 1, 3, 7, 14, and 28 days of cryopreservation or methanol fixation. The RIN scores obtained were compared with the control RIN scores (Figure 1B).

We found that the RNA integrity of ACME HS-dissociated cells was well-preserved during cryopreservation in 3xSSC* and 10% DMSO over the specified intervals (1, 3, 7, 14, and 28 days), with RIN scores averaging around 6.7 (Figure 1B). This preservation was consistent across adrenocortical tumors, adrenal medullary tumors, thyroid carcinoma, and PitNET, allowing for subsequent scRNA-seq analysis (Figure 1B; Supplementary Figure S1). Additionally, after 6 months of storage, the RNA integrity of cryopreserved ACME HS-dissociated adrenocortical cells was 5.9 (Supplementary Figure S2A). The RNA integrity of dissociated PitNET cells was assessed at only two time points (0 and 1 day) due to the small tissue sample size. The RIN of enzyme-dissociated adrenocortical cells fixed in 80% methanol decreased over time with significant degradation of ribosomal RNA evident in reduced or absent signals for the 18S and 28S peaks (Figure 1C; Supplementary Figure S1).

The RNA integrity of fresh-frozen adrenocortical tumor samples after 1, 3, 7, 14, and 28 days of freezing at −80°C ranged from 9.3 to 9.0, with no discernible patterns in RIN score changes over time (Figure 1D). These RIN scores indicate that the material is suitable for subsequent dissociation using the ACME HS method.

### 3.3 Flow cytometry reveals heterogeneity of ACME HS and enzyme-dissociated endocrine samples

**FIGURE 2:**
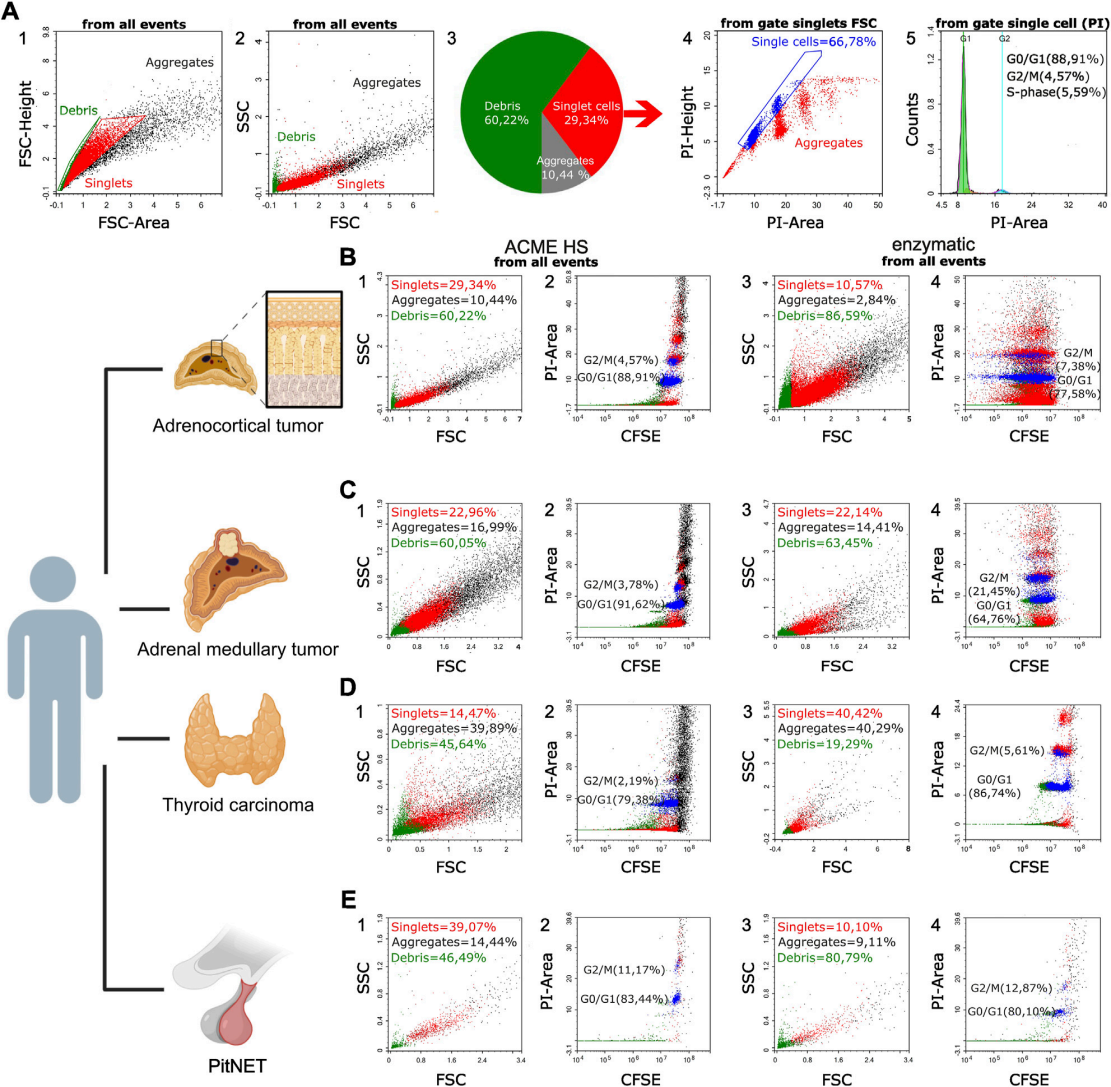
Representative flow cytometry data of the samples prepared by ACME HS and enzymatic dissociation methods. **(A)** Flow cytometry data of the ACME HS dissociated adrenocortical tumor sample (replicate 1). A1. FSC-height/FSC-area dot plot was used to calculate cellular debris, single cells, and cellular aggregates (green events - for debris, red events - for singlets, black events—for aggregates). A2. FSC/SSC dot plot demonstrating the distribution of cells, their aggregates, and cellular debris based on their light-scattering properties. A3. Pie diagram of the debris, singlets and aggregates distribution in the sample. A4. PI-height/PI-area dot plot of singlets used for additional gating of single cells (shown in blue) among nucleated cells and their aggregates (shown in red). A5. DNA histogram from single events showing the cell cycle distribution for all cells in the sample, with percentages of the cell cycle phases (G0/G1, S, G2/M) inserted. **(B–E)** Flow cytometry data for the different samples (replicate 1). B1–B4 – for the adrenocortical tumor; C1–C4 — for the adrenal medullary tumor; D1–D4 — for the thyroid carcinoma; E1–E4 — for PitNET (green events - for debris, red events - for singlets, black events — for aggregates). Index one stands for FSC/SSC dot plots for the samples obtained by the ACME HS protocol. Index two stands for CFSE/PI dot plots for the samples obtained by the ACME HS protocol. Index three stands for FSC/SSC dot plots for the samples obtained by enzymatic dissociation. Index four stands for CFSE/PI dot plots for the samples obtained by enzymatic dissociation. Created with BioRender.com.

We used the flow cytometry method to assess the quality of ACME HS and enzyme-dissociated samples. We compared the amount of debris, aggregates, and single cells obtained by the two protocols of tissue dissociation–ACME HS and enzymatic across different tumor types. We evaluated the ACME-HS method by three independent repeats for each tumor type. It appeared more straightforward to calculate the amount of cellular debris and the number of singlets and aggregates at the FSC-area/FSC-height dot plot (Figure 2A1) than at the FSC/SSC dot plot (Figure 2A2), as the boundary between debris and singlets, and between singlets and their aggregates, is usually poor. We gated out the area of debris (green) as small events with the highest ratio of FSC-height to FSC-area signal. Singlets (red) were selected based on their well-correlated height versus area signal, while aggregates of cells (black) had an increased area signal compared to the height signal. (Figure 2A1; Supplementary Figure S3, A1–H1).

Further, we used a backgating strategy to evaluate the data in other dot plots to correct our subsequent gating for the best separation and calculation of the amount of cellular debris and the number of singlets and aggregates. First, we applied color gating and visualized debris, singlets and aggregates in the FSC/SSC dot plots for all tissues (Figures 2A, 2B1–E1, B3–E3; Supplementary Figure S3, A2–H2). We made sure that debris was located in the lower left area of the dot plot, and the aggregates formed clusters in the upper right area of the FSC/SSC dot plot. Variations in the ratios of singlets and debris across the different tumors studied are expected due to the unique structures and components of these tumor tissues. The highest amounts of singlets were found in adrenal medullary tumor samples, ranging from 15.15% to 54.51%, while the lowest were in thyroid carcinoma samples, ranging from 5.14% to 36.12%. PitNET and adrenocortical tumor samples had average single cell ratios of 28.57% and 27.95%, respectively. Debris levels were consistent across all tissue types, averaging 53.41%. Adrenocortical tumor samples had the lowest aggregate levels, averaging 15.26% compared to other tissues.

Next, we stained the samples with a DNA-binding dye–propidium iodide (PI), to better discriminate the nucleus-contained cells from the nuclear-free debris. We studied the PI-height/PI-area dot plots and checked the positions of debris, singlets, and aggregates among all ungated events including debris and aggregates (Supplementary Figure S3, A3–H3). Then, we gated the single cells (blue) among the nucleated cells and their aggregates (red) (Figure 2A4; Supplementary Figure S3, A4–H4) and analysed DNA histograms from single cells (Figure 2A5; Supplementary Figure S3, A5–H5). In addition, we found that more than 60% of analyzed single cells are located in the G0/G1 cell cycle phase, in all tumor types. Much less cells are located in G2/M and S-phase in all tumor types.

Although our dissociation protocols differed from those that were specifically elaborated for cell cycle analysis and frequently used (Darzynkiewicz, 2011), in most cases, we could resolve various phases of the cell cycle (G0/G1, S, G2/M) by mean fluorescence intensity (MFI) per cell in DNA histograms: DNA content in G2/M phase was as expected, two times more than in G0/G1 as shown in Supplementary Figure S3, A5–H5.

To discriminate the nature of the debris, we stained the samples with 5,6-carboxyfluorescein diacetate succinimidyl ester (CFSE). This dye is frequently used in flow cytometric protocols for live cells labeling due to its ability to bind to intracellular molecules, primarily to amine groups. In addition to its role in viable cell staining, CFSE can trace dying cells in composite samples (Dumitriu et al., 2001). As shown in Figures 2B2–E2, B4–E4, most green events matching debris turned out to be CFSE-positive and PI-negative, which suggested that the debris was generally nuclear-free. By comparing the frequency of debris and aggregates (Supplementary Figure S3, A1–H1) and analyzing dot plots, we suggest that both ACME HS and enzymatic methods induced a relatively similar number of aggregates and debris. Despite the large amount of debris and aggregates, which was expected, we observed a sufficient number of single cells in our samples obtained by the ACME HS and enzymatic dissociation protocols (Figures 2A, B1–E1, B3–E3; Supplementary Figure S3, A2–H2).

### 3.4 ACME HS demonstrates consistency with enzymatic method and keep advantages over nuclei isolation protocol

For comparative analyses, we selected 41 human endocrine tumor samples (Supplementary Table S1). We obtained 107,875 cells and nuclei isolated from adrenocortical tumors (n = 12), 94,807 cells and nuclei from adrenal medullary tumors (n = 15), 41,418 cells and nuclei from PitNETs (n = 9), and 60.365 cells from thyroid carcinomas (n = 5).

**FIGURE 3:**
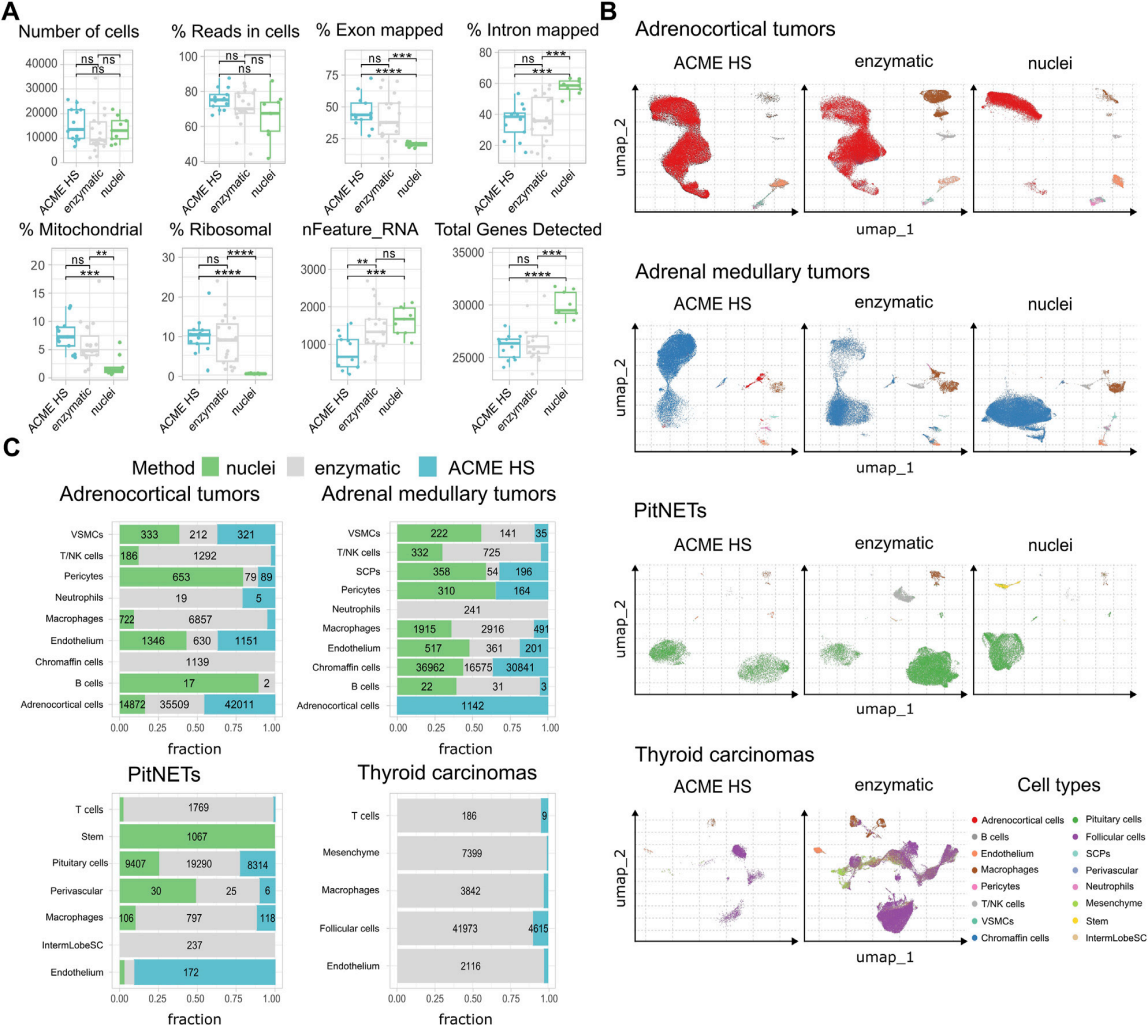
ACME HS demonstrates consistency with the enzymatic protocol and keeps advantages over the nuclei isolation. **(A)** Standard single-cell sample features (number of cells; read the cells%; exon/intron mapped; mitochondrial, ribosomal, nFeature_RNa; and total genes) of ACME HS, enzymatic and nuclei samples. Statistical differences estimated by the Wilcoxon rank-sum test: **** (0.0001 < *p* < 0.001), *** (*p* < 0.001), ns - not significant–*p* > 0.05. **(B)** Major cell type compositions among preparation methods, namely, adrenocortical, chromaffin, pituitary, and thyroid follicular cells. **(C)** Fractions of defined cells identified by different dissociation methods (ACME HS, enzymatic, and nuclei) for each tissue type–adrenocortical tumor, adrenal medullary tumor, thyroid carcinoma, and PitNET. The diagram does not indicate the number of cells representing less than 5% of the total number.

First, we examined the summary statistics for the generated single cell gene libraries (Figure 3A; Supplementary Figure S4). We found that the quality of the ACME HS dataset was almost identical to the enzymatic and nuclei datasets, obtained from ACME HS-dissociated and enzyme-dissociated whole cells, and isolated nuclei, respectively. Some differences in ribosomal and mitochondrial gene expression levels between nuclei and whole cells obtained by ACME HS and enzymatic dissociation methods were confirmed directly, as well as the exon/intron alignment ratio (Figure 3A). Nuclei-based data showed clear advantages in terms of total genes detected, as expected. At the same time, there were no significant differences in quality parameters, namely, the total number of cells, reads in cells for all samples (Figure 3A). We observed the patterns mentioned earlier in the four tissues with different dissociation methods (Supplementary Figure S4).

**FIGURE 4:**
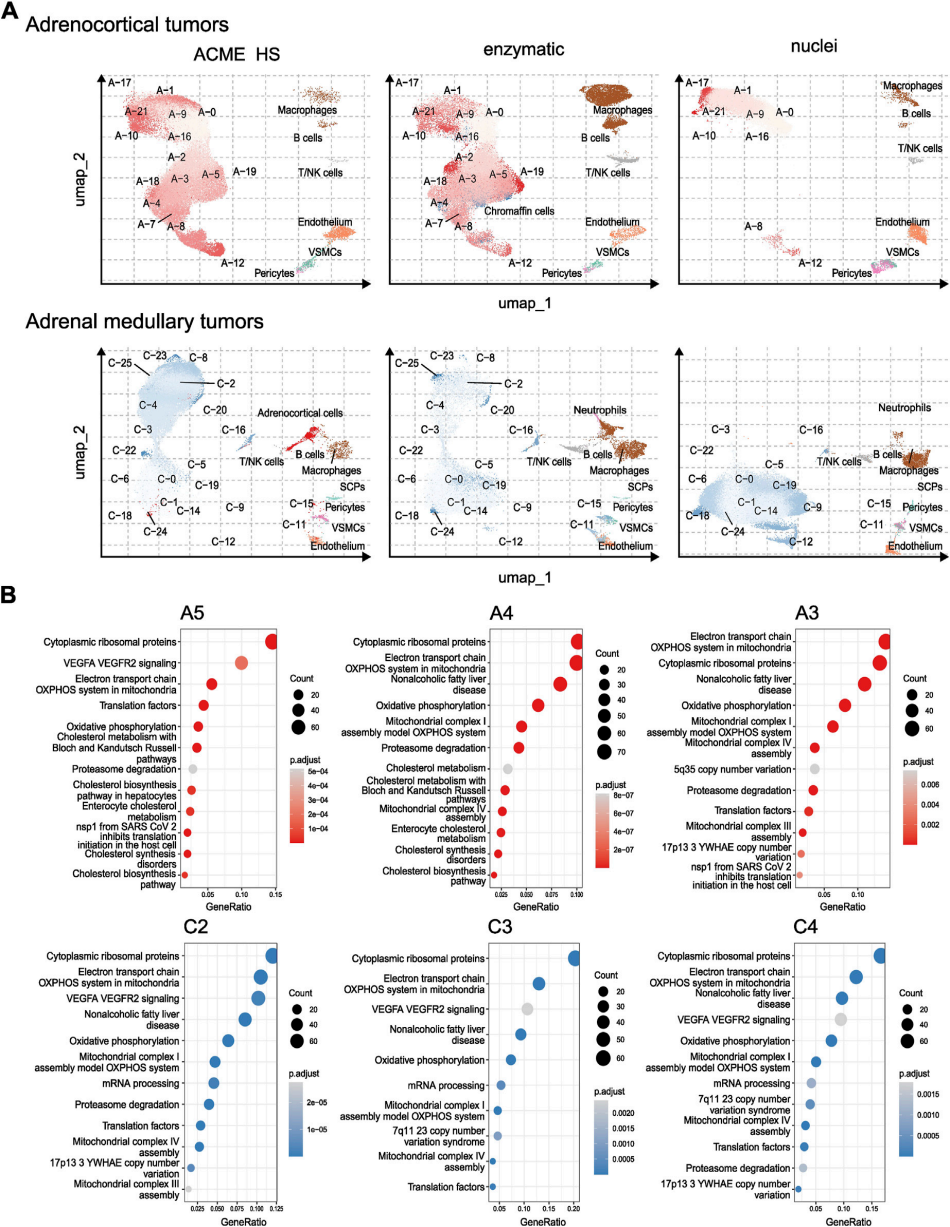
Heterogeneity and functional characterization of cell clusters specific for ACME HS, enzymatic and nuclei datasets **(A)** Visualization of the major cell subpopulations and states for adrenocortical and chromaffin cells. Adrenocortical and chromaffin cells were segregated by cell clustering applied on integrated data sets. Cells segregated into small clusters (<100 cells) were combined into separate minor groups and excluded from the analysis. **(B)** Differentially expressed genes (upregulated in selected clusters compared to all the rest cells, logFC >0.25 and FDR <0.05) were calculated for each cluster within major cell types and analyzed by using wiki pathways as a reference database for main clusters of adrenocortical subpopulations—A-3, A-4, A-5, and chromaffin cells—C-2, C-3, C-4.

To compare the representation of distinct cell types and states between the enzyme, nuclei, and ACME HS datasets, we used Seurat (Hao et al., 2023) to generate integrated embeddings and annotations for each tissue type. ACME HS successfully integrated into enzymatic and nuclei datasets (Supplementary Figure S5). The major cell types were consistently defined and integrated across all three methods. In all four types of tissues, the ACME HS data retained tissue-specific cells, namely, adrenocortical, chromaffin, pituitary, thyroid follicular cells, and other nonspecific cells (Figures 3B,C). The heterogeneity of the major cell populations (adrenocortical, chromaffin, thyroid follicular, and pituitary cells) was assessed by further clustering of the integrated cells (Figure 4A; Supplementary Figure S6). Minor subclusters (<100 cells) were excluded from the analysis. We found that most subclusters (A-2, A-3, A-5, A-4, A-7, A-18, and A-19) were lost for the adrenocortical samples in the nuclei datasets, unlike in the ACME HS and enzyme datasets.

Loss of numerous subclusters was also observed in the nuclei datasets of the chromaffin and pituitary samples (C-2, C-4, C-8, C-20, C-23, and C-25 and P-1, P-2, P-3, P-5, P-10, P-15, P-16, and P-17, respectively). However, some subclusters were more enriched in the nuclei datasets for chromaffin samples (С-0, С-1, С-5, С-6, С-14, and С-19) (Figure 4A; Supplementary Figure S6).

While some cell subpopulation variability is expected due to the individual tissue conditions, the most apparent difference is determined for nuclei-based samples. The main clusters of adrenocortical subpopulations–A-3, A-4, A-5, and chromaffin cells–C-2 C-3 C-4 enriched in oxidative phosphorylation, electron transport chain, ribosomal, mitochondrial, and mRNA processing genes are missing from nuclear datasets as opposed to ACME HS and enzymatic samples (Figure 4B). Because of the low cell numbers obtained from thyroid carcinoma samples with ACME HS-dissociation, comparisons of major thyroid follicular cell population clusters obtained with ACME HS and enzymatic dissociation methods were not possible for these samples.

### 3.5 ACME HS-dissociated cells maintain the expression of key tissue-specific genes

**FIGURE 5:**
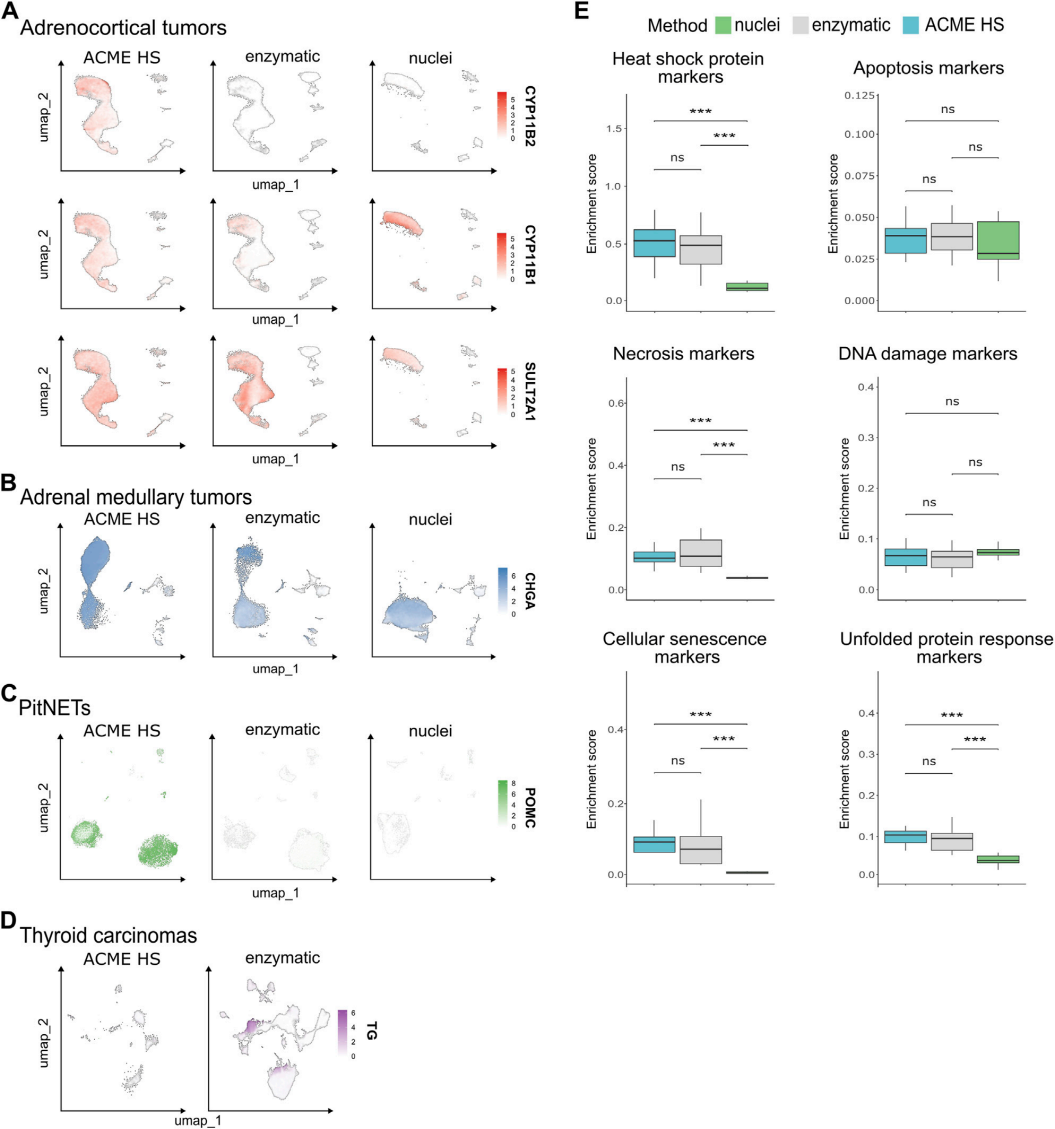
Expression of tissue-specific genes in nuclei and whole cells obtained by ACME HS and enzymatic dissociation methods. **(A)** Key tissue-specific gene expression according to UMAP visualization, namely, *CYP11B2, CYP11B1*, and *SULT2A1* for adrenocortical tumors; **(B)** *CHGA* for adrenal medullary tumors; **(C)** *POMC* for PitNETs; **(D)** *TG* for thyroid carcinoma samples. **(E)** The distribution of the enrichment scores of heat shock, apoptosis, necrosis, DNA damage, cell senescence, and unfolded protein response signatures across preparation methods of the datasets obtained from adrenocortical tumor, adrenal medullary tumor, thyroid carcinoma, and PitNET samples (combined dataset, n = 41).

We next examined whether ACME HS dissociation was able to preserve the expression of tissue-specific marker genes in all four tissue samples. In total, 107.875 cells were obtained from adrenocortical tumor samples (n = 12) and integrated. Adrenocortical cells accounted for 85.6% of the annotated cell types for (Figure 3C). These cells expressed key literature-derived marker genes that identify adrenal cortex zones: *CYP11B2* (van de Wiel et al., 2022) for zona glomerulosa, *CYP11B1* (Duparc et al., 2022) for zona fasciculata, *CYP17A1*, *SULT2A1* (Janšáková et al., 2020), and *CYB5A* (Nakamura et al., 2011) for zona reticularis. These genes were detected in all sample groups regardless of the extraction method, except for *CYP11B2*, which was not detected in the enzymatic and nuclei samples (Figure 5A; Supplementary Figure S7A). Similar results were obtained for the adrenal medullary tumor (n = 15) and PitNET samples (n = 9) with 94.807 (89% chromaffin cells) and 41.418 (79.6% pituitary cells) integrated cells, respectively (Figure 3C). Correspondingly, these cells expressed key marker genes, such as *CHGA*, *SYP* (Mete et al., 2022), *DBH*, *PNMT* (Konosu-Fukaya et al., 2018) (Figure 5B; Supplementary Figure S7B) and *POMC* (only in the ACME HS datasets), with the exception of *GH1* and *POU1F1* in the ACME HS datasets (Figure 5C; Supplementary Figure S7C). Although thyroid follicular cells represented the majority (95.2%) of the 60.365 cells in thyroid gland samples (n = 5), ACME HS-dissociated cells exhibited almost no expression of key markers such as *TG* and *TSHR* (Lee et al., 2015; Liu et al., 2022) (Figure 5D; Supplementary Figure S7D).

Then, we analysed a panel of the top genes specific for adrenocortical, chromaffin, thyroid follicular, and pituitary cells (Supplementary Figure S7E). It turned out that each dissociation method allows to estimate the expression signatures of different genes that have a little overlap in ACME HS, enzymatic, and nuclear samples, with the exception of PitNETs.

### 3.6 Difference in the expression of stress- and apoptosis-associated genes in the ACME HS, enzyme, and nuclei datasets

Since dissociation and preservation techniques can induce cellular stress as evidenced by changes at the transcriptomic level, we examined the expression of key markers of stress and cell death. Specifically, markers associated with apoptosis, necrosis, cellular senescence, DNA damage, heat shock, and the unfolded protein response (UPR) were assessed in the ACME HS (n = 13), enzyme (n = 19), and nuclei (n = 9) datasets for all tissue samples (Figure 5E; Supplementary Figure S8; Supplementary Tables S1, 2).

We found no differences in the expression levels of genes related to apoptosis in any of the datasets. The necrosis gene signature showed no differences between the ACME HS and enzymatic samples but was lower in the nuclei samples. Nevertheless, the most significant expression of *TNF* (tumor necrosis factor) was observed in adrenocortical tumors and thyroid carcinomas in ACME HS and nuclei samples (Supplementary Table S2). The DNA damage gene signature exhibited minimal variations among all three methods. In addition, the heat shock protein signature showed minimal differences between the ACME HS and enzymatic samples but was significantly lower for nuclei samples. The UPR signature did not differ between ACME HS and enzymatic samples but was significantly lower for nuclei samples. In particular, *ERN2,* a UPR marker, was highly expressed in adrenal medullary tumor and PitNET datasets obtained by ACME HS and nuclei isolation methods, in adrenocortical tumors by enzymatic digestion, and in thyroid carcinomas by the ACME HS method. In addition, the cellular senescence signatures also showed minimal differences between the ACME HS and enzymatic samples. *IL1B* was highly expressed in adrenocortical and medullary tumor samples obtained from the ACME HS and nuclei datasets, while *IL6* was highly expressed in all tissues compared with the PitNET datasets obtained by the enzymatic digestion method. Another important senescence marker, *CDKN2B,* was highly expressed in adrenal medullary tumor samples obtained by nuclei isolation as well as in thyroid carcinoma samples obtained by an enzymatic approach. Senescence markers *HMGA1* and *UBB* were highly expressed in the nuclei datasets for adrenocortical and medullary tumor samples (Supplementary Table S2).

### 3.7 RNA velocity estimates of the individual cells accurately recapitulate the transcriptional dynamics in the ACME HS and enzyme datasets

**FIGURE 6:**
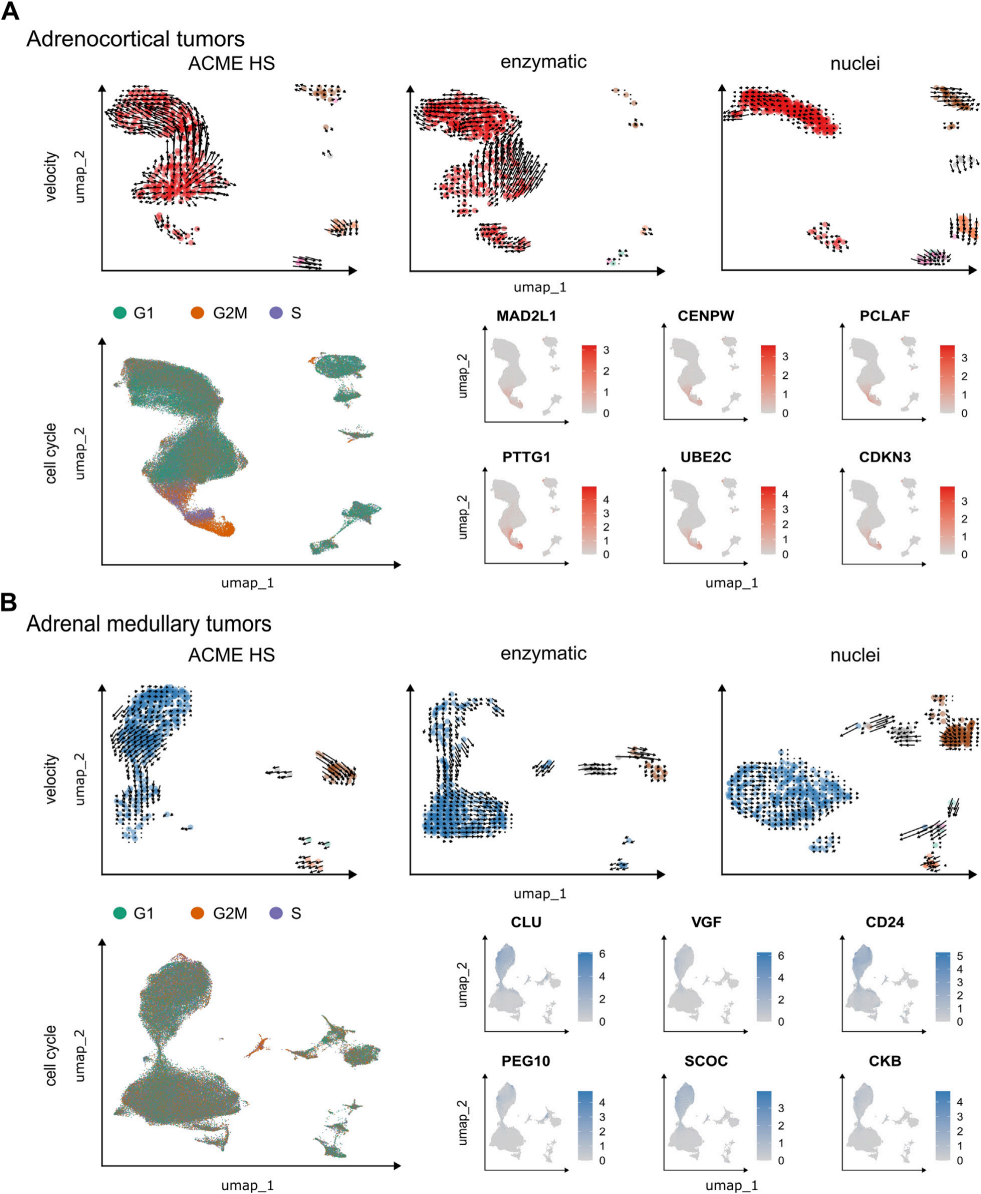
Velocity and cell cycle of the ACME HS, enzymatic and nuclei datasets. **(A, B)** Velocity and cell cycle estimation for adrenocortical and adrenal medullary tumor datasets, respectively. Velocity was performed for individual samples (n = 1) for each method, and cell cycle estimation was performed for adrenocortical (n = 12) and adrenal medullary tumors (n = 15). Examples of differentially expressed (DE) genes associated with cell cycle control are shown on the individual embeddings. DE analysis was conducted for specific clusters within major cell types, such as A-7, A-8, and A-12 and C-16, C-19, and C-23 (Figure 4).

Next, we performed velocity analysis for individual samples to demonstrate the consistency between the ACME HS (n = 1) and enzymatic (n = 1) protocols together with their advantages over the nuclei isolation method (n = 1). For adrenocortical (Figure 6A) and chromaffin cells (Figure 6B), we identified similar velocity directionalities from ACME HS and enzymatic-specific clusters towards cell populations commonly shared between methods. This was confirmed with significance assessments (Supplementary Figure S9). The RNA velocity recapitulated the transcriptional dynamics within these datasets, including the general movement of the differentiating adrenocortical and chromaffin cells, as well as movement towards and away from the intermediate differentiation state. The velocity also captured the cell cycle dynamics involved in cell differentiation.

We observed G2M and S phase cells at velocity start point in ACME HS and enzymatic specific clusters as well as differential expression of cell cycle controlling and neuroendocrine tumor proliferation genes–*MAD2L1, CENPW, PCLAF, PTTG1, UBE2C*, *CDKN3* for adrenocortical cells and *CLU, VGF, CD24, PEG10, SCOC*, *CKB* for chromaffin cells in all samples. Identified genes demonstrated significant differential expression with p.adjusted (FDR) < 1E-298 in proliferating cells. These genes were found in clusters A-7, A-8, and A-12 in the adrenocortical tumor samples, and in clusters C-16, C-19, and C-23 in the adrenal medullary tumor samples (Figure 4; Supplementary Table S3). Cells expressing these genes were distributed between the S and G2/M phases of the cell cycle, with virtually no G1 phase present (Figure 6; Supplementary Figure S10; and Supplementary Table S4). This indicates that they are potential progenitors of adrenocortical and chromaffin cells. Although proliferating cells in PitNETs were determined as a separate cell cluster, we did not find a clear-cut pattern in RNA velocity and the cell cycle (Supplementary Figure S11). Due to low single cell numbers obtained from thyroid carcinoma samples, a clear-cut pattern in RNA velocity was not possible for these samples.

## 4 Discussion

In this study, we present an optimized ACME HS dissociation technique for the effective isolation of single cells from fresh-frozen human tissues. In the real-life setting of clinical research centers, the utilization of fresh tissues is rather complicated and frequently disruptive for cells that are to be further analysed using scRNA-seq (see *Introduction for details*). Consequently, the utilization of fresh-frozen tissues collected through biobanking represents a robust alternative, facilitating both prospective analyses and retrospective studies.

ACME HS, which employs simultaneous acetic acid-based dissociation and methanol-based fixation “snaps” the transcriptional profiles of individual cells at the very beginning of the procedure, thereby eliminating the global transcriptome changes associated with the action of dissociation enzymes and displacement of cells from their original tissue context/microenvironment. Yet, another important point is that the ACME HS allows for the recovery of high-integrity RNA even following cryopreservation of fixed cell suspensions. Finally, in contrast to single nuclei isolation techniques, ACME HS preserves the cytoplasm of cells, yielding a dramatically better representation of mature mRNAs.

Here, we demonstrated that ACME HS is suitable and efficient technique for isolating of the high-quality single cells from difficult-to-dissociate complex tissues with high lipid content and large areas of fibrosis and calcinosis, the tissues typically posing significant challenges for enzymatic digestion. We have successfully obtained 304,465 cells from 41 endocrine neoplasms, namely, adrenal medullary tumors, adrenocortical tumors, thyroid follicular cell-derived carcinomas and the pituitary-derived neuroendocrine tumors by ACME HS, enzymatic, and nuclei isolation methods.

One of the key optimization points as compared to the original ACME technique was the use of the high-salt 3xSSC* washing buffer instead of PBS. The main rationale behind this point is that, under physiological ionic strength, RNAses may be reactivated during the rehydration, thereby dramatically diminishing the yield and integrity of mRNA and ribosomes. In that, 3xSSC* supplemented with DTT and RNase inhibitor dehydrates the cells and blocks the activity of RNAses (Chen et al., 2018), allowing for an efficient preservation of nucleic acids inside the cells.

Using flow cytometry, we were able to provide evidence for obtaining of sufficient cell numbers employing ACME HS technique, with standard DNA histograms and accurately determined cell cycle phases further confirming the proper processing of our samples. The degree of cellular debris and subG1-fragments may be attributed to the freeze-thawing of the samples during both the enzymatic and ACME HS protocols and does not compromise our conclusions. In fact, this correlates with the results of the study of the ACME-dissociation method performed by García-Castro et al. where their study showed that despite the large quantity of cellular debris they got, which was higher than the debris count we got in all our samples, the ACME method enables the investigation of cell type diversity in a given tissue (García-Castro et al., 2021).

We further assessed the cellular stress responses associated with the different sample processing techniques. Since the stress response genes are known to be activated upon the proteolytic tissue dissociation at 37°C, we expected the major differences in the expression profiles there of between ACME HS/single nuclei isolation performed under the ice-cold conditions vs. enzymatic dissociation protocol performed at 37°C. However, despite our initial considerations, the total contributions of the stress signatures (heat shock, necrosis, cellular senescence, and UPR) in enzymatic and ACME HS dissociation protocols of tumor tissues were essentially the same, with exposure to collagenase and membrane rupture during methanol incubation (causing loss of cytoplasmic mRNA) being deduced as the major stress factors in enzymatic and ACME HS protocols, respectively. The nuclei isolation protocol significantly outperformed both enzymatic and the ACME HS dissociation methods in terms of the reduced stress responses identified in scRNA/snRNA-Seq profiles. In addition, nuclei-based data offers benefits in gene detection and intronic ratios. By capturing pre-mRNA and non-spliced transcripts, which are often diminished or processed in the cytoplasm, this approach allows for a wider array of transcripts, including immature RNA. As a result, it achieves a greater total gene count and a higher intronic ratio compared to conventional cytoplasmic RNA methods. Both protocols for the isolation of single cell suspensions performed significantly better than isolation of single nuclei in the majority of the other comparisons in our study. However, this specific issue may be highly relevant in studies, where minimizing of sample processing-associated stress responses and/or enrichment of sequencing data with intronic sequences in nuclear immature RNAs are of critical importance, dictating the choose of isolation of single nuclei instead of single cells’ isolation in these cases.

Going to the whole-transcriptome level, we were able to successfully integrate the ACME HS, enzyme, and nuclei datasets, further integrating them with the reference scRNA-Seq profiles of the cognate normal tissues. We examined all of the acquired datasets (ACME HS, enzymatic, nuclei) to evaluate the heterogeneity of the major cell populations (adrenocortical, chromaffin, thyroid follicular, and pituitary neuroendocrine cells) for all four tissues studied. Overall, we demonstrated a comparable representation of the major cell types, subpopulations, and functional states in ACME HS and enzymatic methods, while the single nuclei-based protocol performed significantly much worse. Our data thus corroborate previous observations on the principal differences of scRNA vs. snRNA profiles, particularly in terms of cytoplasm-associated signatures, including those associated with cellular metabolism (Gaedcke et al., 2022), protein synthesis and mRNA processing (Santiago et al., 2023; Bakken et al., 2018), the processes being particularly important for tumorigenesis (Venit et al., 2023; Zhang et al., 2021), with a proper representation thereof being critical for obtaining of the biologically relevant data in the studies of human neoplastic diseases.

Finally, we performed the velocity analysis and assessment of the activity of cell cycle markers to demonstrate that nuclei-based data were largely depleted from the information on putative differentiation directions, intron-retention events, as well as cell cycle phases connectivity. Again, the data obtained from both single cell dissociation protocols were fairly consistent, implying thereof as preferable approaches for studying cell differentiation, clonal evolution in cancer and intron-retention events.

In summary, we optimized and employed the ACME HS technique for the scRNA analysis of human tissues derived from various endocrine neoplasms. We clearly demonstrated that scRNA profiling of single cell suspensions obtained using ACME HS and enzymatic methods significantly outperformed snRNA profiling in terms of marker gene expression analysis and tumorigenesis while demonstrating in-between comparable performances in the majority of implemented analyses. Additionally, the modified ACME protocol allows for an extra-option of successful cryopreservation of dissociated/fixed cells without sacrificing the mRNA yield and integrity. To our knowledge, this is the first report on successful implementation of the ACME HS technique in primary human tissues, and we believe that this protocol should significantly promote the scRNA studies in humans that are to be explicitly compliant with the real-life infrastructure and logistics of the surgical care centers.

## Data Availability

The datasets presented in this study can be found in online repositories. The names of the repository/repositories and accession number(s) can be found below: https://www.ncbi.nlm.nih.gov/geo/, GSE263784. https://www.ncbi.nlm.nih.gov/geo/, GSE264080. https://www.ncbi.nlm.nih.gov/geo/, GSE263061.
