# Supplementary material for: Comparative evaluation of ACetic - MEthanol high salt dissociation approach for single-cell transcriptomics of frozen human tissues
Source: Front Cell Dev Biol. 2025 Jan 7;12:1469955. doi: 10.3389/fcell.2024.1469955 (PMC11748064; doi:10.3389/fcell.2024.1469955)
Supplement: Supplementary file 5 [file DataSheet1.docx]

Supplementary Material

**Comparative evaluation framework of ACetic - MEthanol High Salt dissociation approach for single-cell transcriptomics of frozen human tissues.**

**Marina Utkina** ^1^†*, **Anastasia Shcherbakova** ^1^†*, Ruslan Deviatiiarov^1,2,3^, Alina Ryabova^1^, Marina Loguinova^1^, Valentin Trofimov^1^, Anna Kuznetsova^1^, Mikhail Petropavlovskiy^1^, Rustam Salimkhanov^1^, Denis Maksimov^4^, Eugene Albert^4^, Alexandra Golubeva^1^, Walaa Asaad^1^, Lilia Urusova^1^, Ekaterina Bondarenko^1^, Anastasia Lapshina^1^, Alexandra Shutova^1^, Dmitry Beltsevich^1^, Oleg Gusev^1,2,3,5^, Larisa Dzeranova^1^, Galina Melnichenko^1^, Ildar Minniakhmetov^1^, Ivan Dedov^1^, Natalya Mokrysheva^1^, **Sergey Popov^1^*****.

† These authors contributed equally to this work and share first authorship

**** (all three are corresponding authors)***

****Correspondence:* Marina Utkina** [mv.utkina@yandex.ru](mailto:mv.utkina@yandex.ru)

**Anastasia Shcherbakova** [nastya.shcherbakova1@gmail.com](mailto:nastya.shcherbakova1@gmail.com)

**Sergey Popov** [swpopov73@gmail.com](mailto:swpopov73@gmail.com)


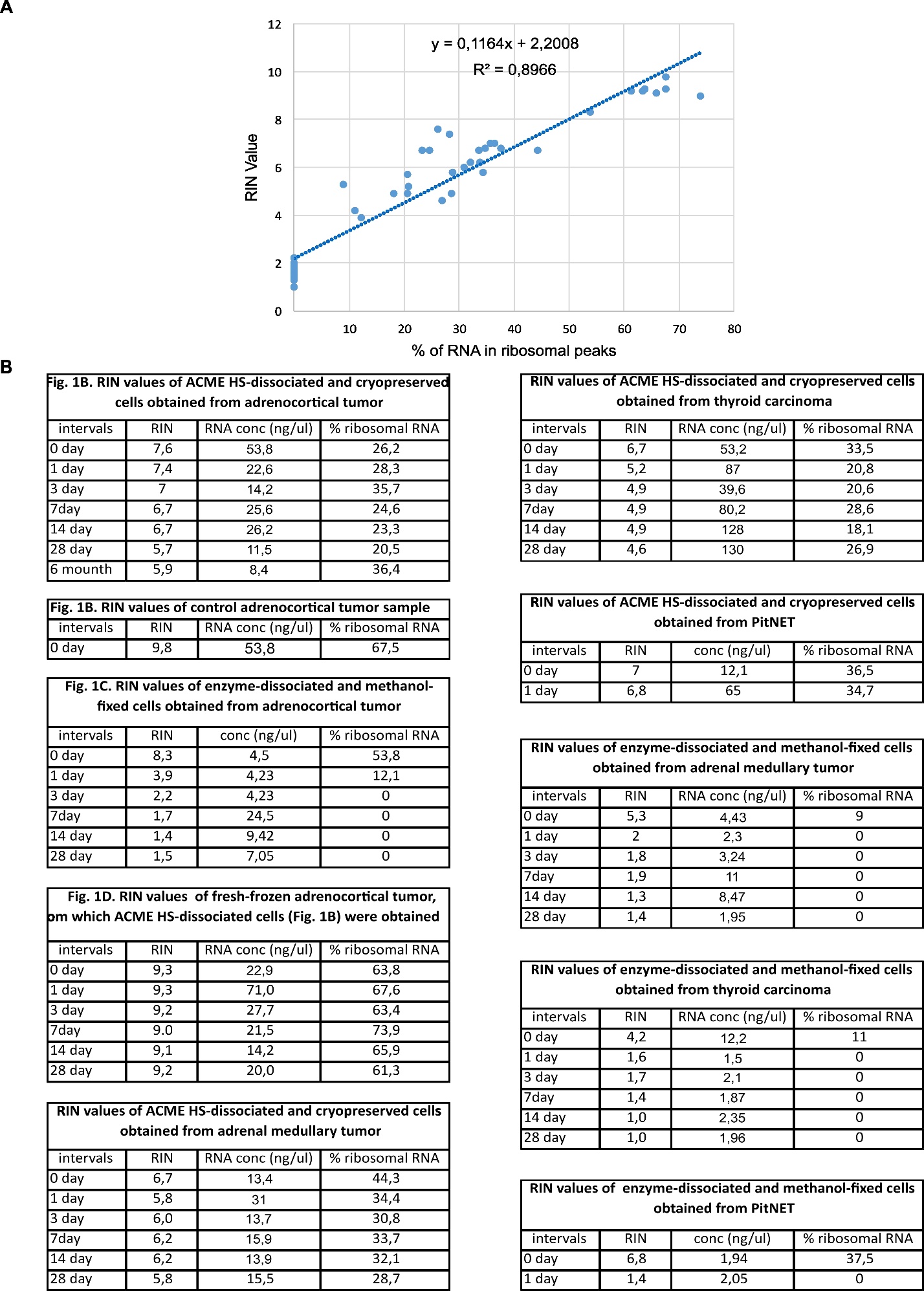


**Supplementary Figure 1. RNA integrity and quantification.**

**A**. Correlative analysis of RIN values and the % area of the two ribosomal bands compared to the total. The linear correlation is indicated on the graph. **B.** Tables containing the individual values extracted from the RNA samples displayed in Figure 1B, 1C, 1D and RNA cell samples of adrenocortical tumor, adrenal medullary tumor, thyroid carcinoma, and PitNET.


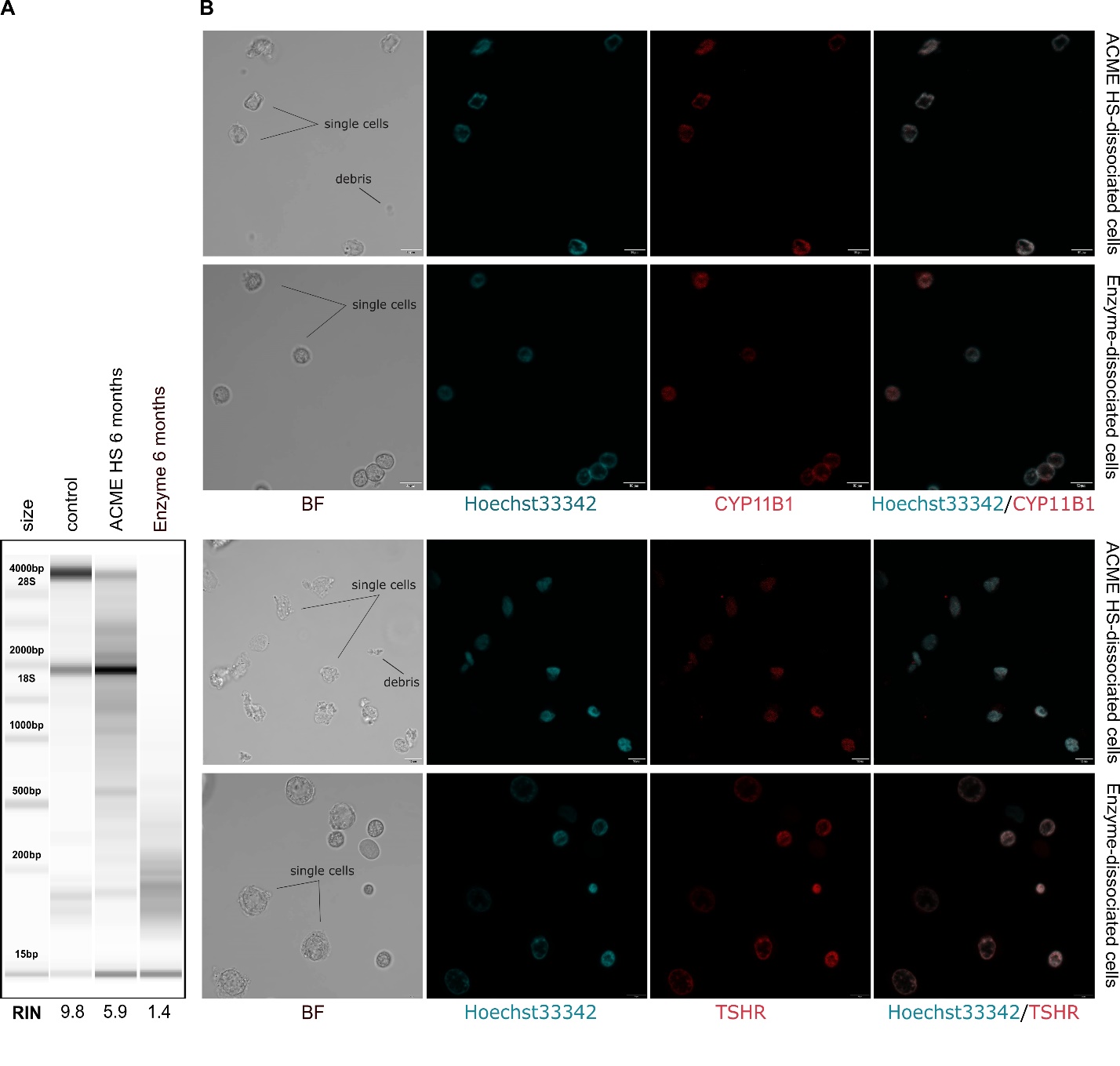


**Supplementary Figure 2. Comparison of storage and morphology of ACME HS and enzyme-dissociated cells.**

**A.** Gel image of isolated total RNA from cryopreserved ACME HS-dissociated and methanol-fixed enzyme-dissociated adrenocortical cells after six months of freezing at -80°C. **B**. Bright field (BF) and confocal fluorescence microscopy images of ACME HS and enzyme-dissociated adrenocortical and thyroid follicular cells stained with anti-CYP11B1 (red), anti-TSHR (red) antibody, respectively, and Hoechst 33342.


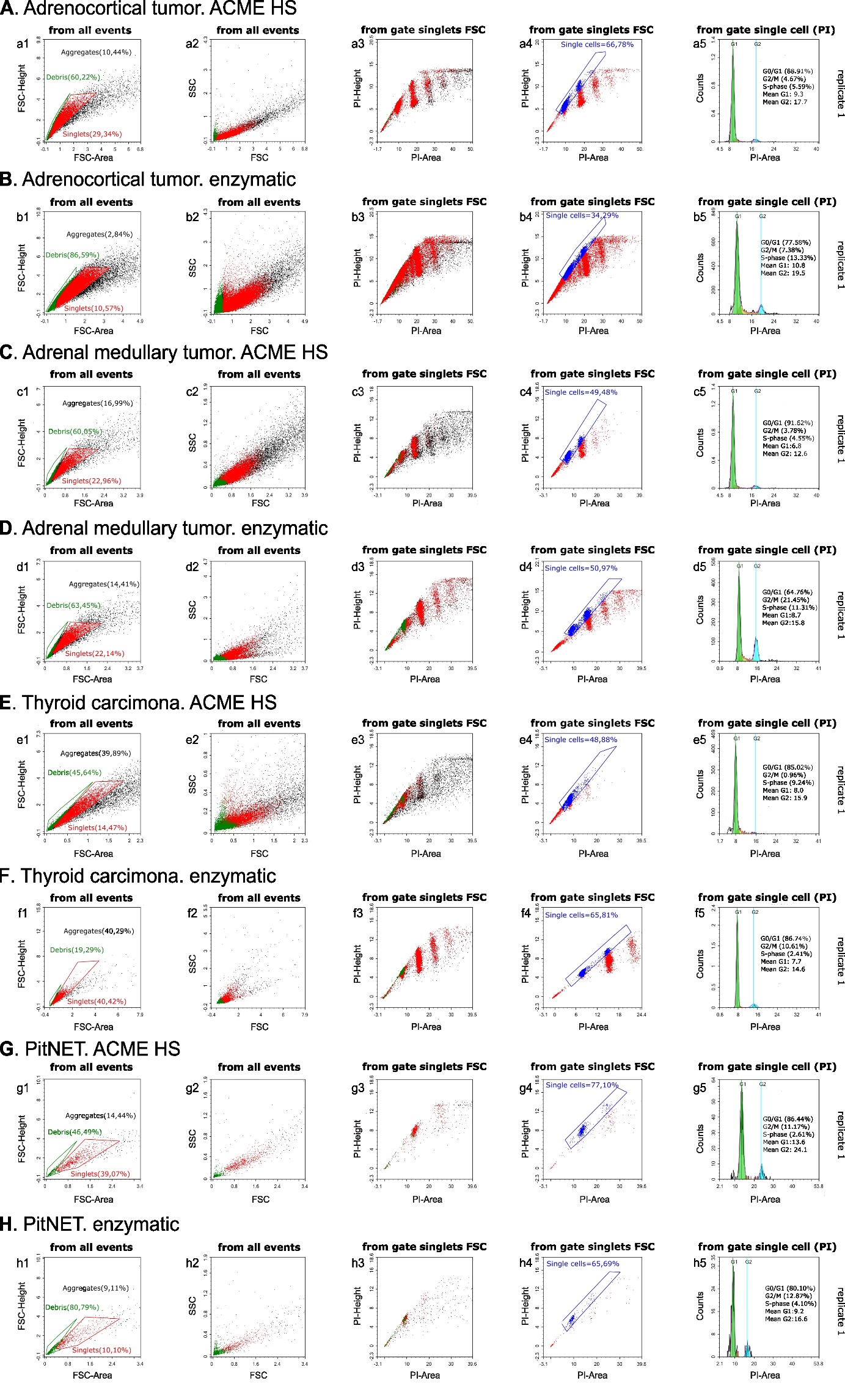

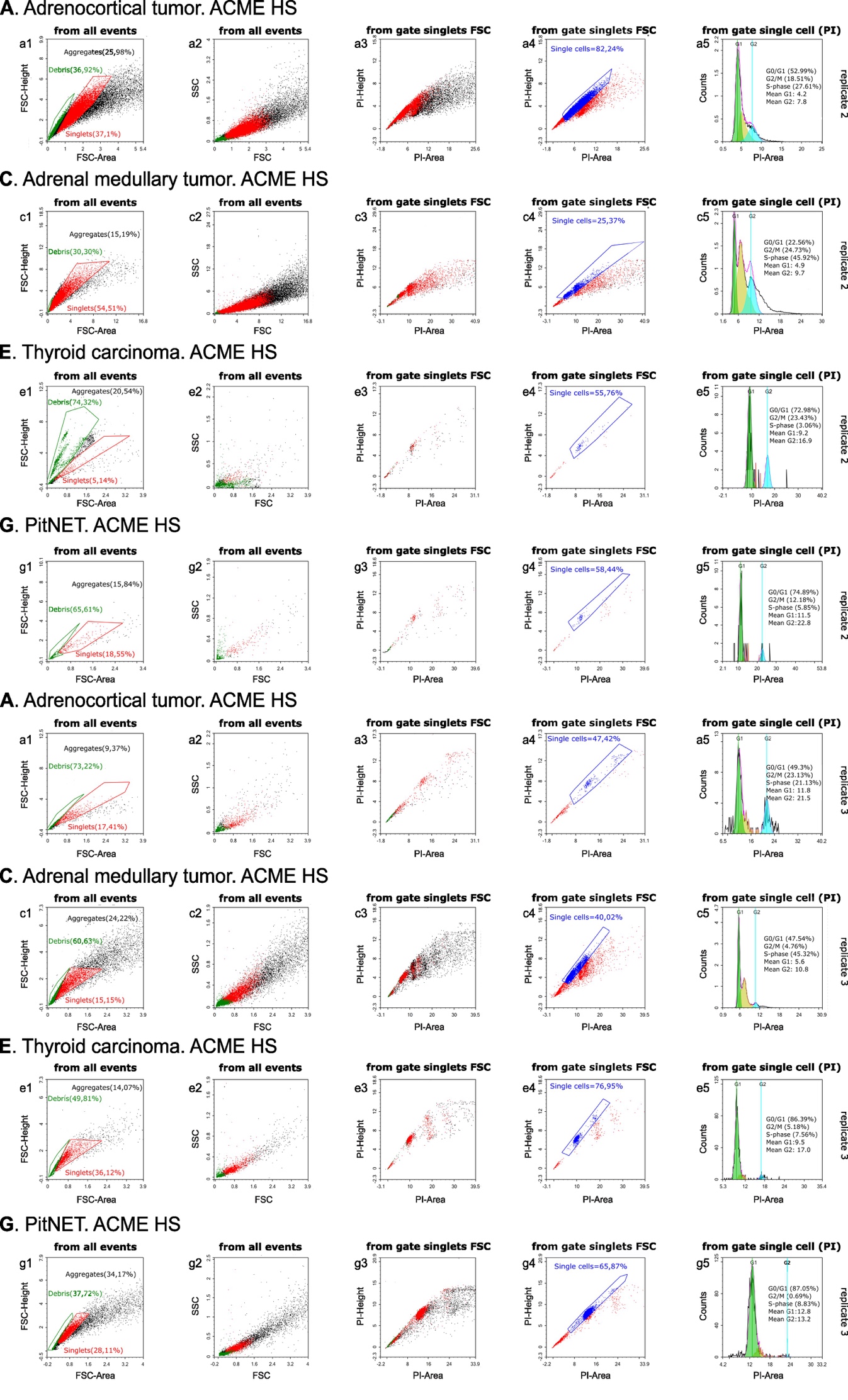


**Supplementary Figure 3. Flow cytometry data of the different samples prepared through ACME HS method or enzymatic digestion**.

**A1-H1.** FSC-height/FSC-area dot plots are used to calculate cellular debris, single cells, and cellular aggregates (green events - for debris, red events - for singlets, and black events – for aggregates). **A2-H2.** FSC/SSC dot plots demonstrate the distribution of cells, their aggregates and cellular debris based on their light-scattering properties. **A3-H3**. PI-height/PI-area dot plots from all ungated events were used for additional location assessment for debris, singlets, and aggregates. **A4-H4**.  PI-height/PI-area dot plots from singlets used for additional gating of single events (shown in blue) among nucleated cells and their aggregates (shown in red). **A5-H5.** DNA histograms from single events showing cell cycle distribution for all cells in the sample, with percentages of the cell cycle phases (G0/G1, S, G2/M) and mean fluorescence intensity for G0/G1 and G2/M phases inserted. The ACME HS method was performed in 3 replicates for each tissue.


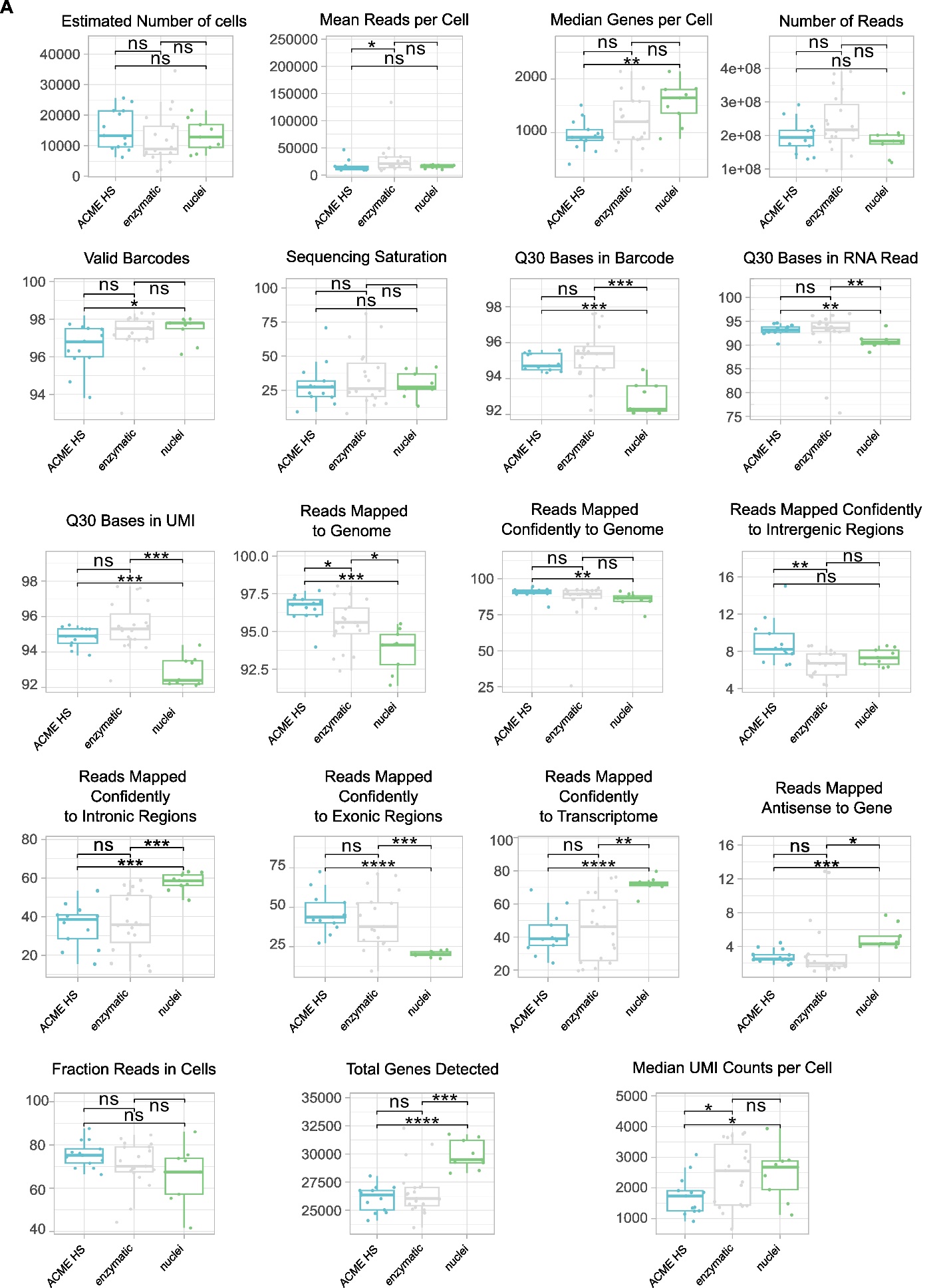


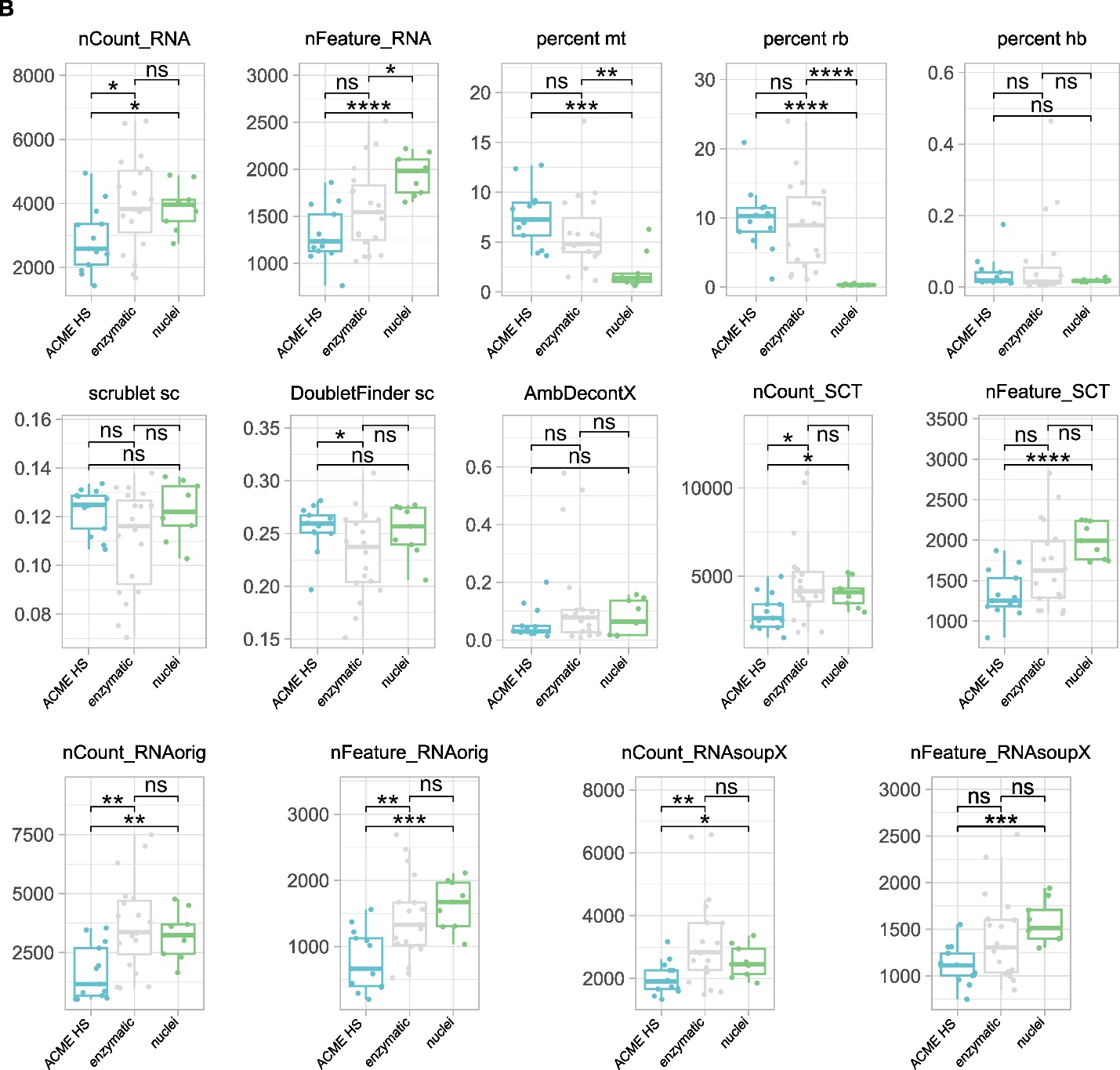


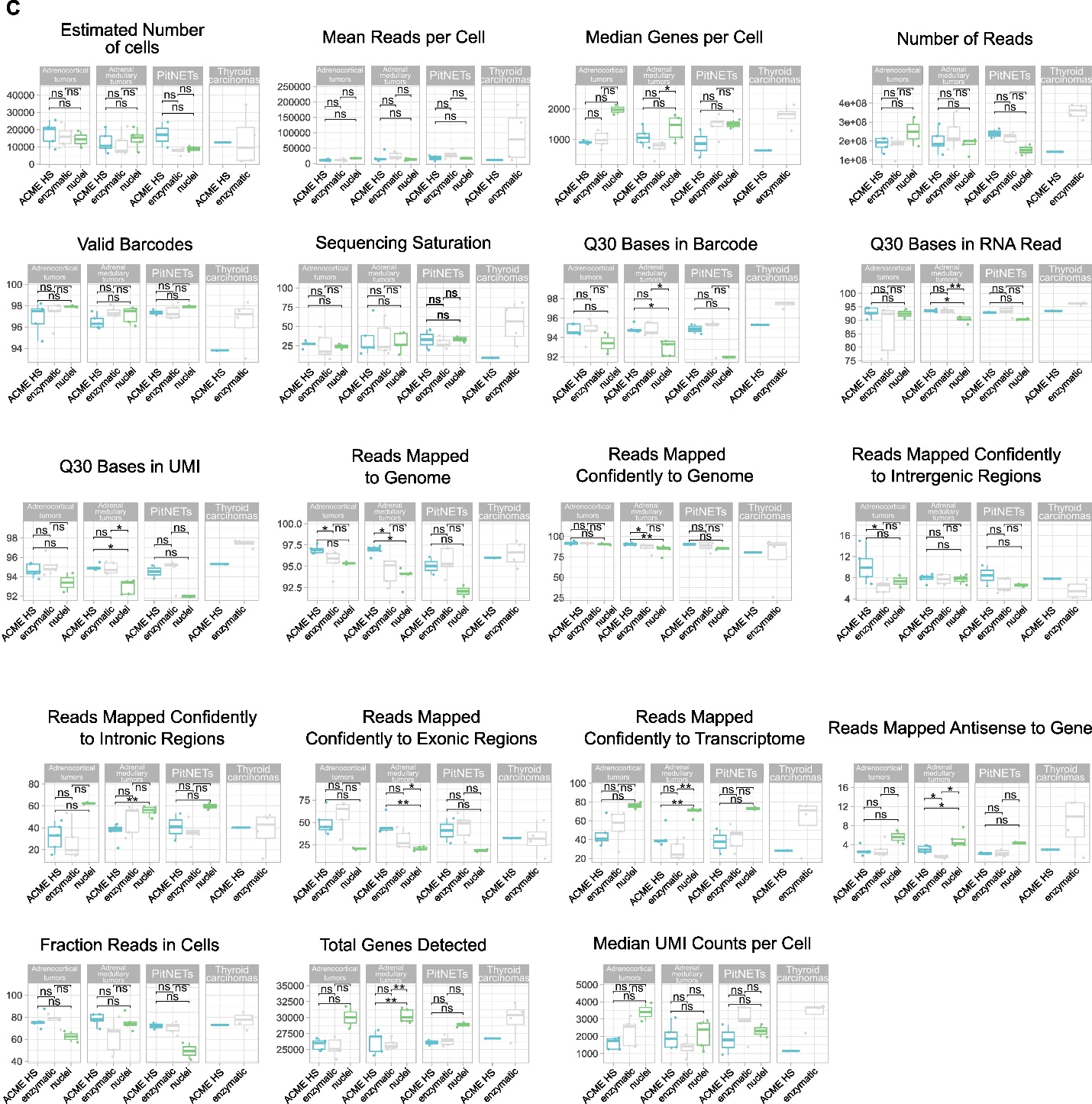


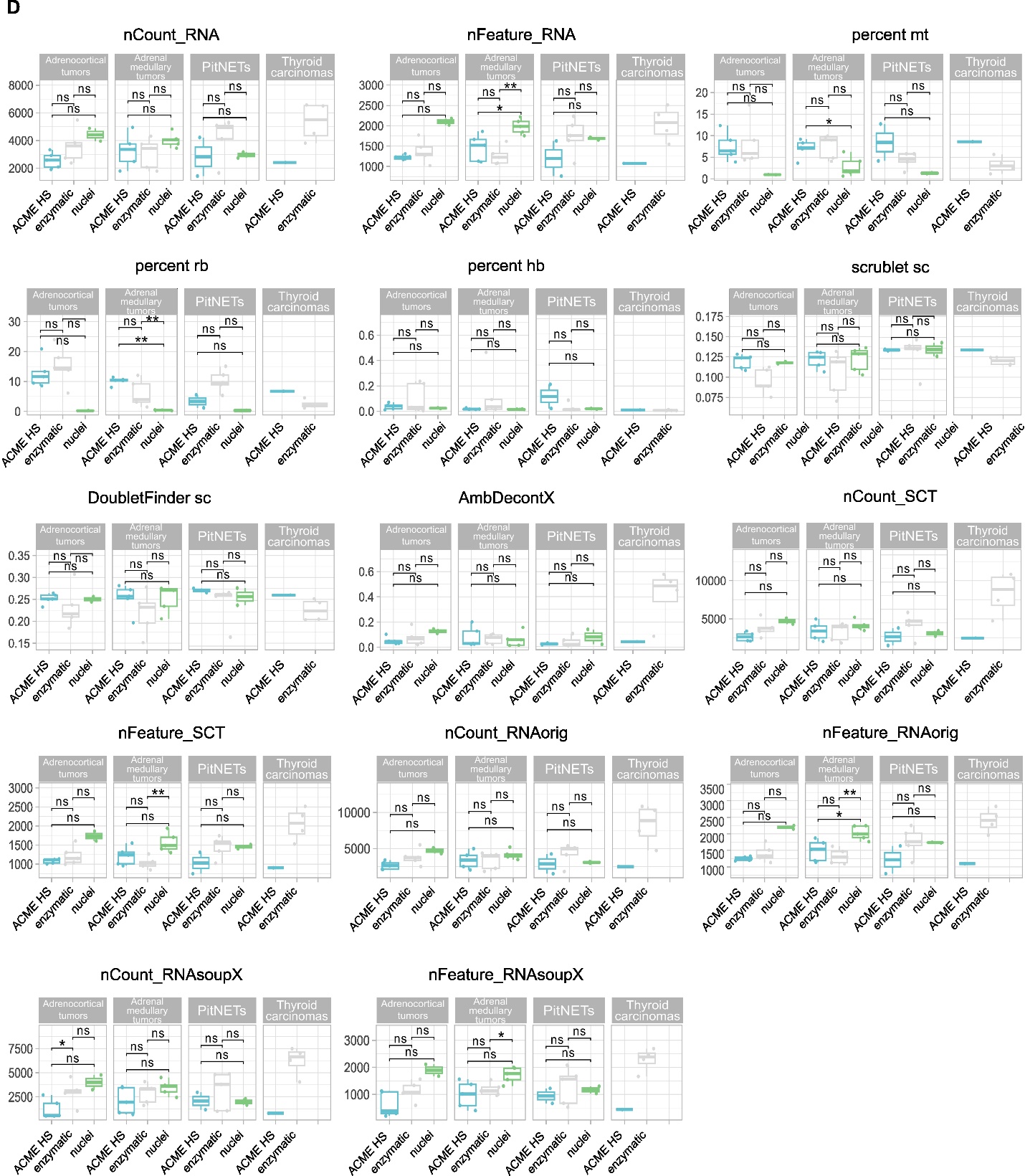


**Supplementary Figure 4. Single-cell sample preparation methods comparison**.

**A**, **B**. Comparison of the basic sample features calculated by the Cellranger pipeline. **C.** Standard single-cell sample features comparisons (number of reads, expressed genes, doublets, etc.) for ACME HS, enzyme and nuclei isolation methods. **D.** Standard single-cell sample features comparisons of all four tissues (adrenocortical tumors, adrenal medullary tumors, thyroid carcinomas, and PitNETs) for each dissociation method. Wilcoxon rank sum used for statistics calculation: **** (0.0001 < p < 0.001), *** (p < 0.001), ** (0.001 < p < 0.01), * (0.01 < p < 0.05), ns - not significant – p > 0.05.


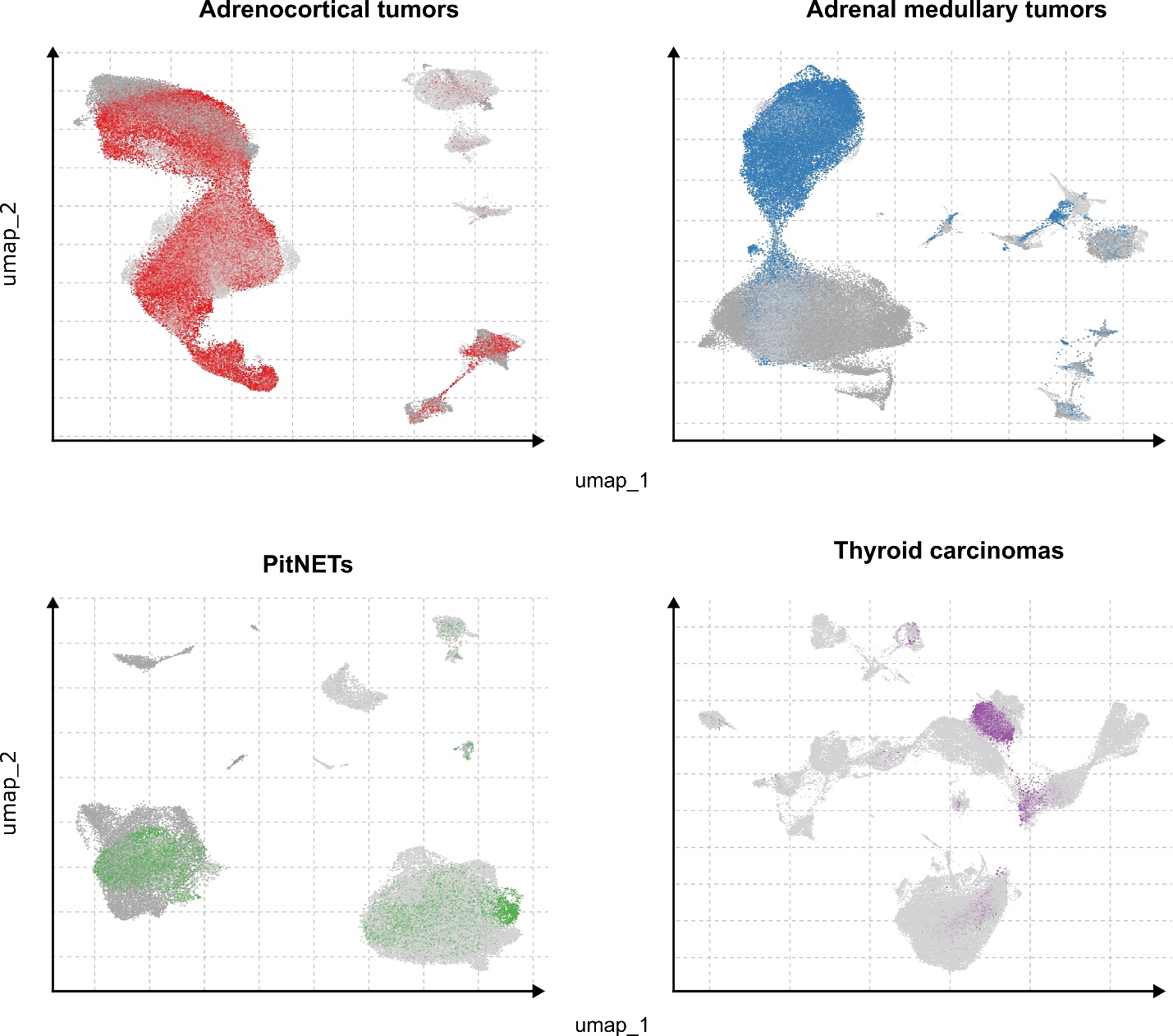


**Supplementary Figure 5. Alignment of the cells derived by ACME HS with enzymatic and nuclei data sets.**

ACME HS cells colored red, blue, green and purple for adrenocortical, chromaffin, thyroid follicular, and pituitary cells, respectively. Cells derived by enzymatic and nuclei methods colored gray.


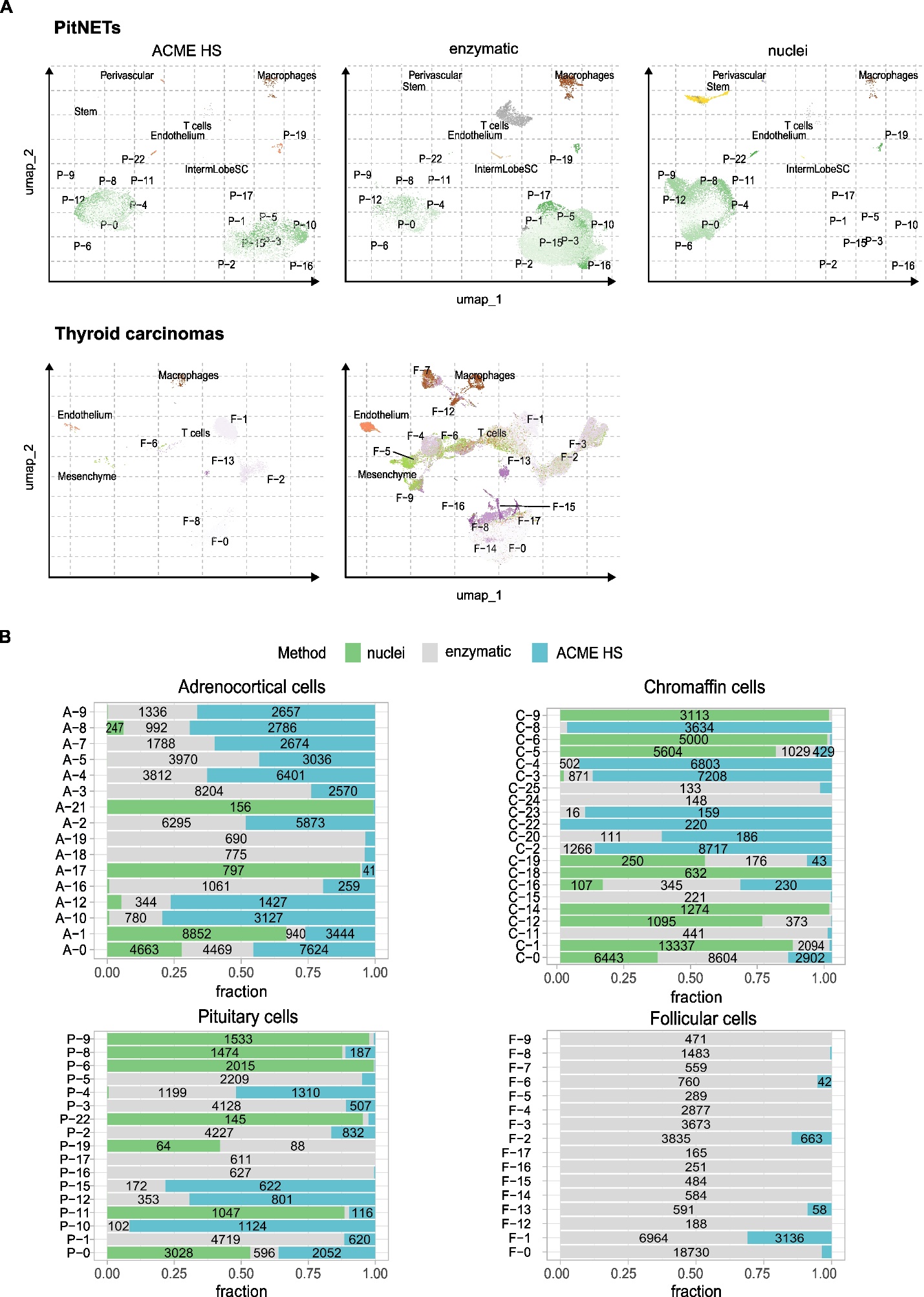


**Supplementary Figure 6. Heterogeneity of major cell types in tumor samples by different methods.**

**A**. Visualization of the major cell subpopulations and states for PitNET and thyroid carcinoma samples. Pituitary (P) and follicular (F) cells were segregated by cell clustering applied on integrated datasets. **B**. Fractions of defined subpopulations and states identified by different methods. Cells segregated into small clusters (<100 cells) were combined into separate minor groups and excluded from the analysis. The diagram does not indicate the number of cells representing less than 5% of the total number.


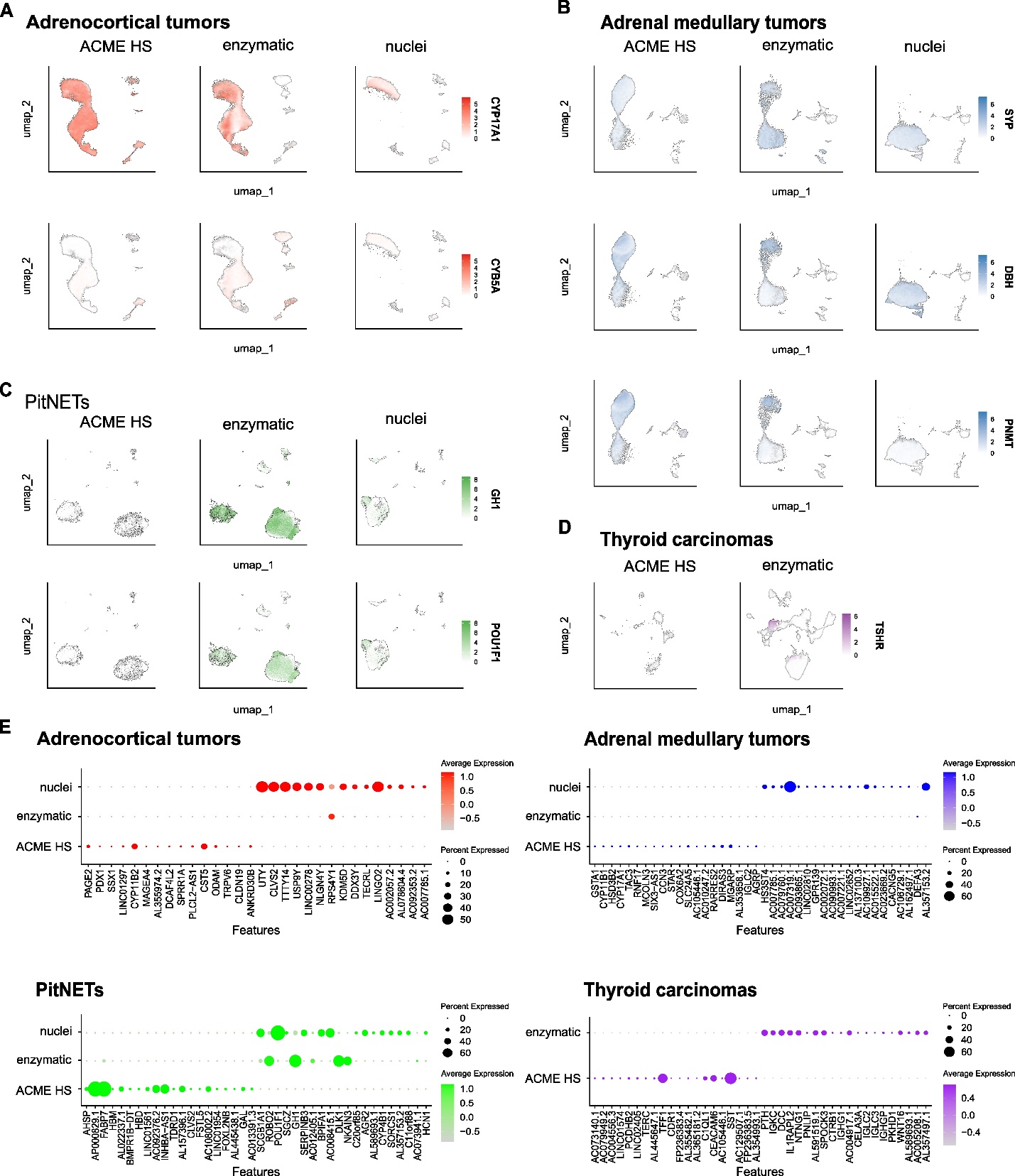


**Supplementary Figure 7. Top gene examples contributing to the differences between tested methods.**

**A.** Gene expression changes for selected gene examples are visualized on UMAP namely, *CYP17A1* and *CYB5A* for adrenocortical tumor; **B –** *SYP, DBH,* and *PNMT* for adrenal medullary tumor; **C –** *GH1* and *POU1F1* for PitNET; **D –** *TSHR* for thyroid carcinoma samples. **c.** Top differentially expressed genes (n=20) shown on the dotplots. **E.** For the differential expression analysis ACME HS was compared against enzymatic and nuclei methods.


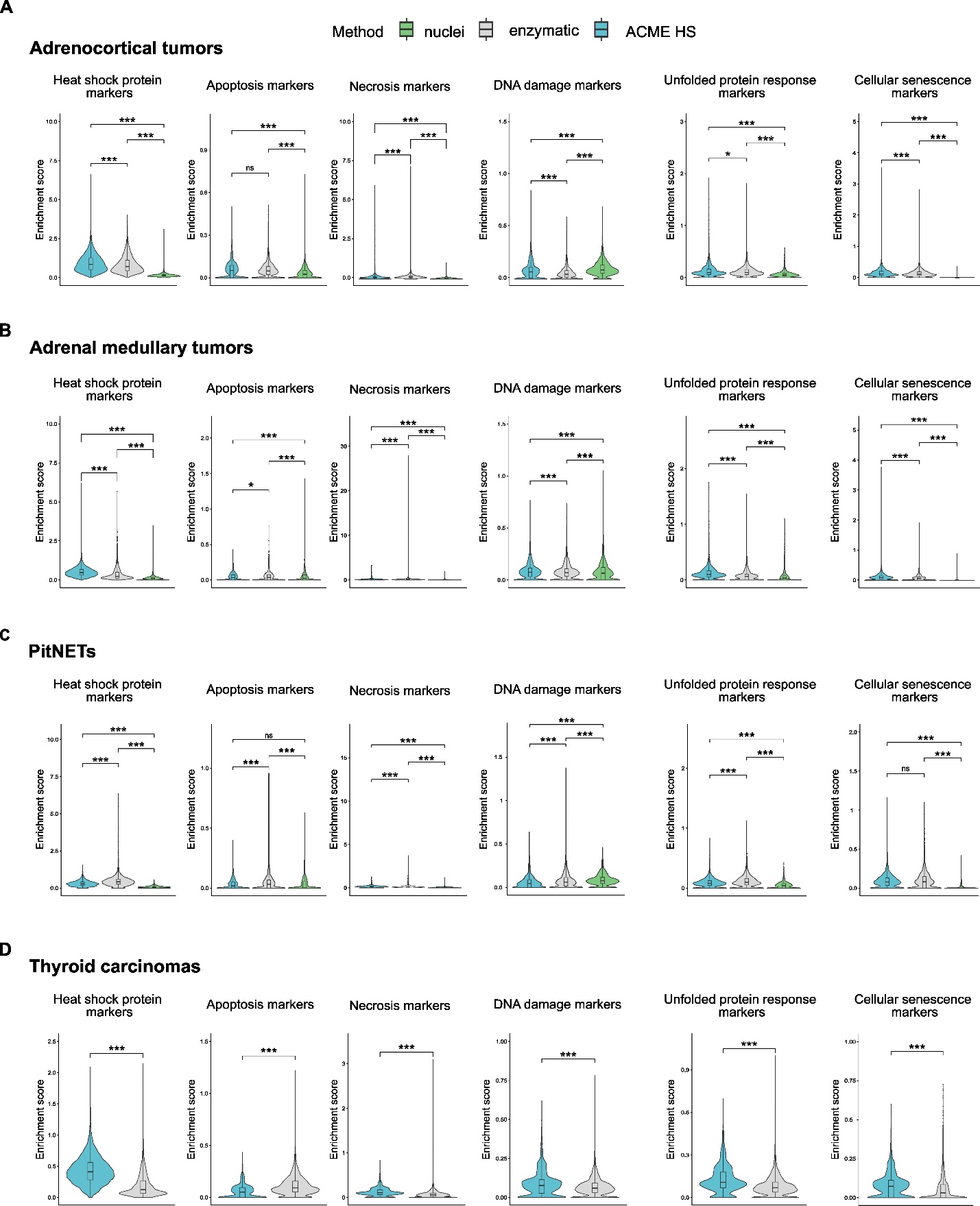


**Supplementary Figure 8. Violin plots showing the distribution of the enrichment scores of different signatures across sample preparation methods.**

The boxplots included inside the violin plots summarize the data distribution. Upper and lower sides of the box represent the 1st and 3rd quartiles. The line in the middle corresponds to the median. Lines extend no further than 1.5 the interquartile range. In all cases, statistical significance was tested using a one-tailed Wilcoxon rank-sum test:  *** (*p* < 0.001), * (0.01 < *p* < 0.05), ns - not significant – *p* > 0.05. **A, B, C, D.** The stress signatures for adrenocortical tumor (n=12), adrenal medullary tumor (n=15), thyroid carcinoma (n=8), and PitNET (n=9) samples, respectively.


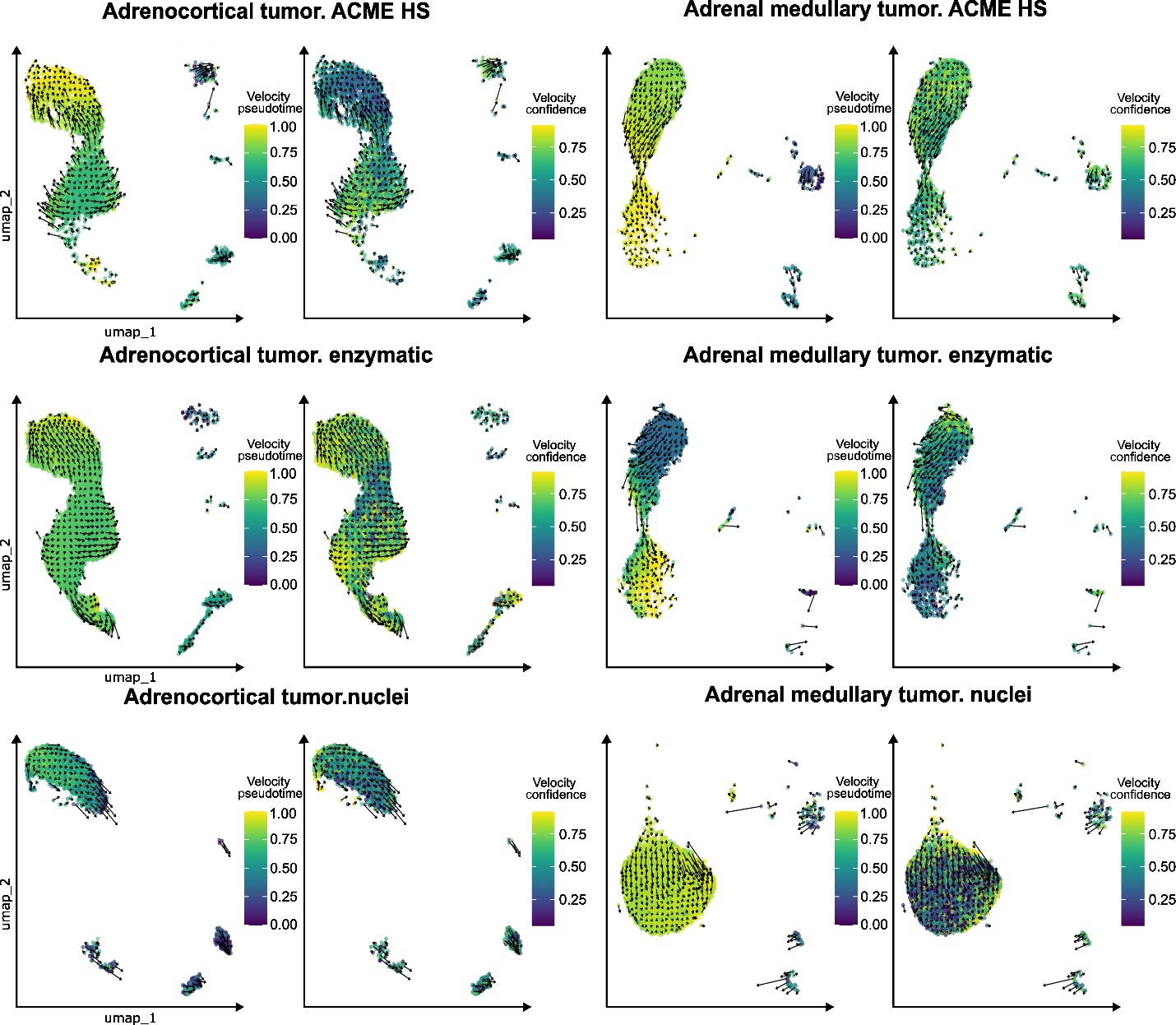


**Supplementary Figure 9. Velocity estimations with pseudotime and confidence metrics on the adrenocortical tumor and adrenal medullary tumor samples.**

Velocity estimation for adrenocortical tumor and adrenal medullary tumor datasets. Analyze was performed for individual samples (n=1) for each method.


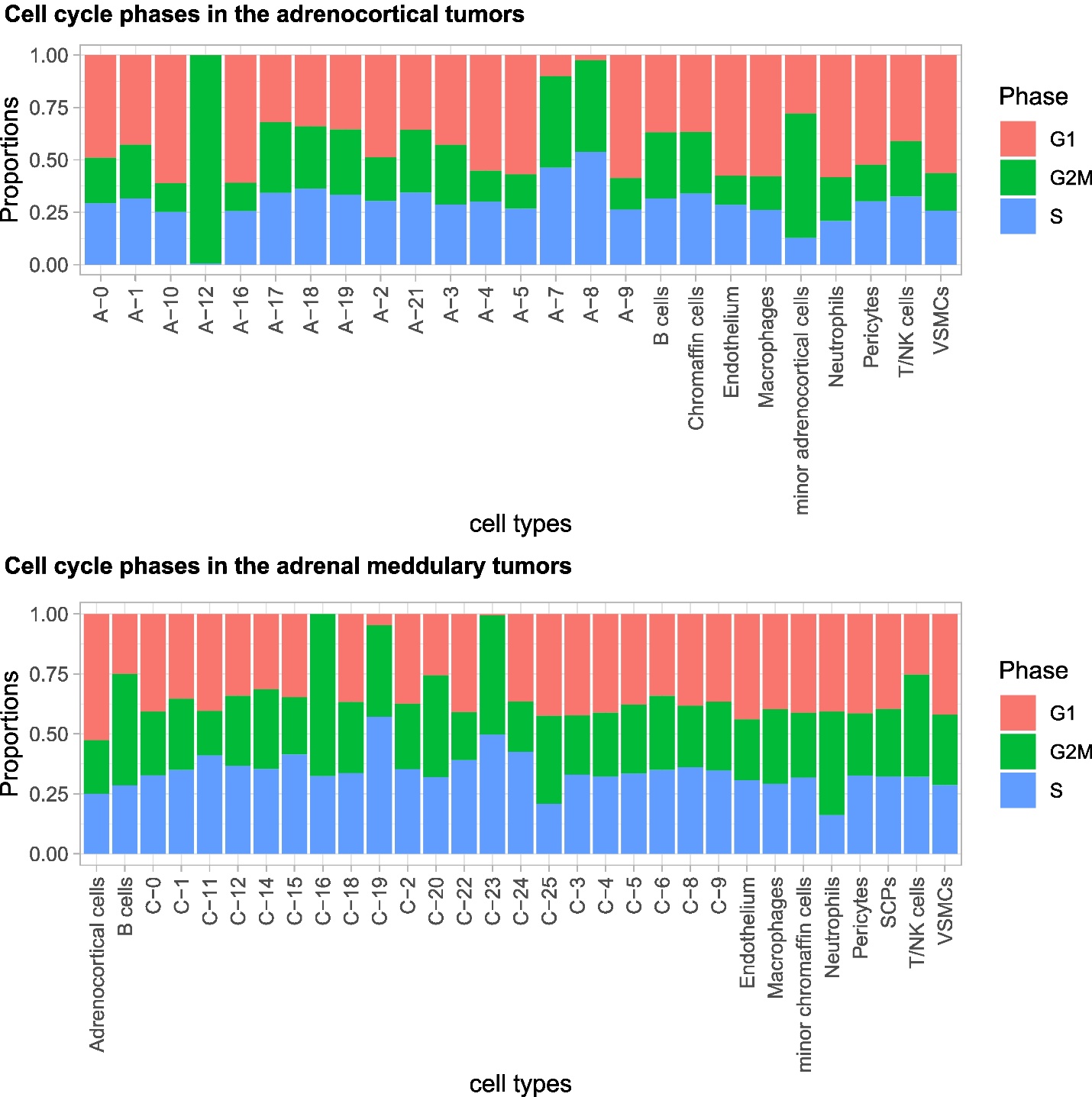


**Supplementary Figure 10. Cell phases fractions per cell types in the adrenocortical tumor and adrenal medullary tumor samples.**

Сell cycle estimation for adrenocortical tumor datasets (n=12) and adrenal medullary tumor datasets (n=15).


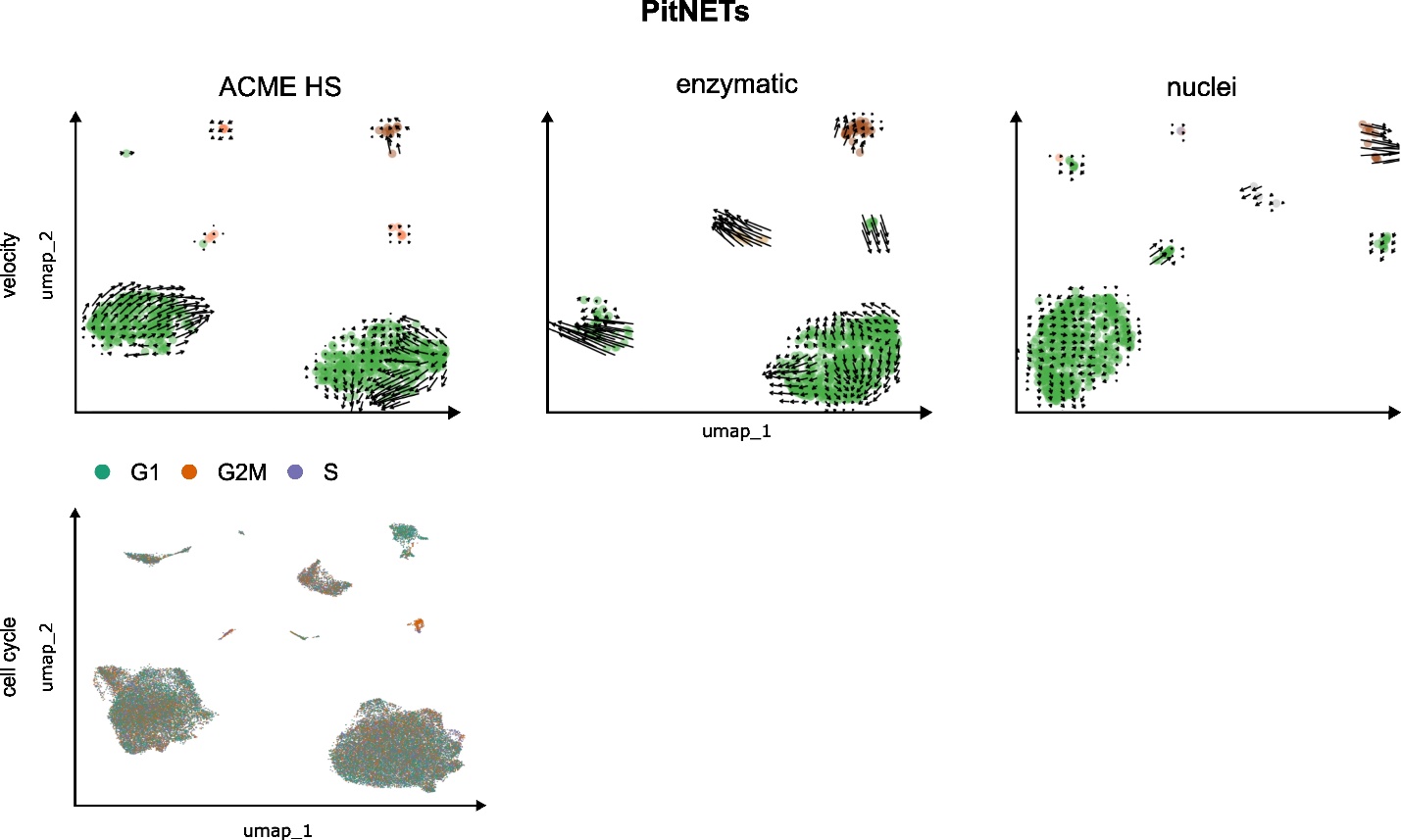


**Supplementary Figure 11. Velocity and cell cycle estimations for PitNET datasets.**

Velocity was performed for individual samples (n=1) for each method, cell cycle estimation for PitNETs (n=9).
